# Primary donor triplet states of Photosystem I and II studied by Q-band pulse ENDOR spectroscopy

**DOI:** 10.1007/s11120-022-00905-y

**Published:** 2022-03-15

**Authors:** Jens Niklas, Alessandro Agostini, Donatella Carbonera, Marilena Di Valentin, Wolfgang Lubitz

**Affiliations:** 1 Max Planck Institute for Chemical Energy Conversion, Stiftstrasse 34-36, 45470 Mülheim an der Ruhr, Germany; 2 Present Address: Chemical Sciences and Engineering Division, Argonne National Laboratory, 9700 S. Cass Ave., Lemont, IL 60439 USA; 3 Department of Chemical Sciences, University of Padova, via Marzolo 1, 35131 Padova, Italy; 4 Biology Centre, Institute of Plant Molecular Biology, Czech Academy of Sciences, Branišovská 31, 370 05 Ceske Budejovice, Czech Republic

**Keywords:** Chlorophyll triplet state, Triplet EPR, ENDOR, Spin density distribution, P680, P700

## Abstract

The photoexcited triplet state of the “primary donors” in the two photosystems of oxygenic photosynthesis has been investigated by means of electron-nuclear double resonance (ENDOR) at Q-band (34 GHz). The data obtained represent the first set of ^1^H hyperfine coupling tensors of the ^3^P700 triplet state in PSI and expand the existing data set for ^3^P680. We achieved an extensive assignment of the observed electron-nuclear hyperfine coupling constants (hfcs) corresponding to the methine α-protons and the methyl group β-protons of the chlorophyll (Chl) macrocycle. The data clearly confirm that in both photosystems the primary donor triplet is located on one specific monomeric Chl at cryogenic temperature. In comparison to previous transient ENDOR and pulse ENDOR experiments at standard X-band (9–10 GHz), the pulse Q-band ENDOR spectra demonstrate both improved signal-to-noise ratio and increased resolution. The observed ENDOR spectra for ^3^P700 and ^3^P680 differ in terms of the intensity loss of lines from specific methyl group protons, which is explained by hindered methyl group rotation produced by binding site effects. Contact analysis of the methyl groups in the PSI crystal structure in combination with the ENDOR analysis of ^3^P700 suggests that the triplet is located on the Chl *a*ʹ (P_A_) in PSI. The results also provide additional evidence for the localization of ^3^P680 on the accessory Chl_D1_ in PSII.

**Supplementary Information:**

The online version contains supplementary material available at 10.1007/s11120-022-00905-y.

## Introduction

Chlorophyll triplet states (^3^Chl) in photosynthetic reaction centers (RCs) are carefully avoided in nature since they are reactive species that can convert ground state triplet molecular oxygen, ^3^O_2_, to singlet oxygen ^1^O_2_, a very dangerous cell poison (Krieger-Liszkay 2004). Hence, in photosynthetic proteins Chl triplet states are typically effectively quenched by carotenoids (Frank and Cogdell 1996; Young et al. 1999; Telfer 2002; Di Valentin et al. 2013; Di Valentin and Carbonera 2017), which are in close contact to Chls with optimized arrangements thereby enabling efficient triplet–triplet energy transfer to the carotenoid and subsequent dissipation of the excess energy by heat. In antenna systems of light harvesting complexes or in the intrinsic antennas of photosynthetic RCs, ^3^Chls are populated by inter-system crossing (ISC) under excess light excitation. In the photosynthetic RCs the process of triplet generation is very different. Intersystem crossing triplet formation of the primary donors in PSI and PSII is typically not observed, since the electron after photoexcitation is rapidly (100 fs–10 ps range) transferred to subsequent cofactors in the electron transfer chain (Brettel 1997; Dekker and Van Grondelle 2000; Prokhorenko and Holzwarth 2000; Shelaev et al. 2010; Mamedov et al. 2015; Duan et al. 2017). Only when forward electron transfer to the first long-lived electron acceptors in RCs is blocked (A_1_ or Pheo in PSI or PSII, respectively), the primary donor triplet state (^3^P) can be generated with high yield (Frank et al. 1979; Budil and Thurnauer 1991; Lubitz et al. 2002). The mechanism involves the conversion of the (singlet) “primary” radical pair (RP) (P700^·+^A_0_^·−^ or P680^·+^Pheo^·−^ in PSI or PSII, respectively) to a (triplet) RP due to g-tensor anisotropy and hyperfine interactions in the two spin-carrying cofactors of the primary pair. Charge recombination from the (triplet) primary RP then leads to the formation of the triplet state of the primary donor (Okamura et al. 1987; Telfer et al. 1988; Setif and Bottin 1989; Budil and Thurnauer 1991; Brettel 1997; Lubitz 2002). Note, that it is not a priori clear on which of the Chls belonging to the RC the triplet state is located; a similar problem exists for the earliest steps of charge separation where different Chls or groups of Chls have been discussed as potential primary donor (Gorka et al. 2021), i.e., the exact nature of the primary donor is not fully understood. In contrast, the location of the longer-lived oxidized “primary donor” P^·+^ has been well established over the years using a combination of various spectroscopic techniques, mutagenesis, isotope labeling and the availability of high-resolution crystal structures (Gorka et al. 2021). We follow the standard practice to name the “primary donor” triplets generated by charge recombination ^3^P700 and ^3^P680 in PSI and PSII, respectively, without implying the assignment to a specific cofactor(s), and also use the term “primary donor” for the long-lived P^·+^ state in both PSI and PSII.

Although the ^3^Chl is not a functional state, it is an important endogenous probe of the electronic structure of Chl molecules in the ET transfer chain, in virtue of the importance of these pigments in the photosynthetic process. In addition, it is important in the context of photoprotection under high light intensities. The electron transfer events in photosynthetic RCs are controlled both by the spatial arrangement of the cofactors and by their electronic properties. The latter are determined by the wave functions and orbital energies of the respective states, modulated by their protein surrounding. The triplet state exhibits two (strongly coupled) unpaired electrons (*S* = 1 state), that are delocalized over the macrocycle(s), thereby probing the electron distribution of both the HOMO and the LUMO, i.e., the frontier orbitals that are also involved in the primary charge separation process. Knowledge of the distribution of the unpaired electrons over the set of frontier orbitals is also a requirement to understand efficient triplet–triplet energy transfer, as already demonstrated for photosynthetic antenna complexes (Di Valentin et al. 2010, 2012; Carbonera et al. 2014a; Cupellini et al. 2016).

The paramagnetic character of triplet states makes electron paramagnetic resonance (EPR) spectroscopy coupled with photoexcitation the most appropriate method for investigating the electronic structure of the pigments in the triplet state (Budil and Thurnauer 1991; Lubitz 2002; Lubitz et al. 2002; Richert et al. 2017; Weber 2018); the “dark” nature of the triplet state makes the application of optical methods more challenging. Thus, time-resolved EPR techniques are frequently used to derive information on the magnitude and orientation of the (traceless) zero-field splitting (ZFS) tensor of the triplet state. The ZFS parameters D and E are sensitive indicators of the spatial extension and symmetry of the triplet exciton, and the spin polarization properties are a fingerprint for the mechanism of formation of the triplet state (Budil and Thurnauer 1991; Lubitz 2002; Lubitz et al. 2002; Richert et al. 2017; Weber 2018). Additional and more specific information about the unpaired electron spin distribution is obtained from the interaction of the triplet state (*S* = 1) with magnetic nuclei of the molecule, i.e., the electron-nuclear hyperfine couplings (hfc). These hfcs reflect the unpaired electron spin density at the respective nucleus (A_iso_) and close to it (A_aniso_) but are in the vast majority of cases not resolved in the time-resolved EPR spectra. To determine the hfcs more advanced methods like Electron Nuclear Double Resonance (ENDOR) experiments are required (Kemple 1979; Gemperle and Schweiger 1991; Möbius and Savitsky 2008; Kulik and Lubitz 2009; Harmer 2016). If hfcs of several nuclei in different parts of the cofactor(s) can be determined and assigned to specific nuclei, the spin density distribution of the triplet state in the respective cofactor(s) is revealed. Pulse ENDOR spectroscopy combined with repetitive laser excitation at low temperatures is well suited since this method takes full advantage of the large electron spin polarization of ^3^P. Furthermore, the large anisotropy of the triplet state ZFS tensor in comparison to the magnitude of the hfcs, allows orientation-selective ENDOR spectroscopy to be performed that provides the orientation of the hfc tensor components relative to the ZFS tensor axes. In addition, the ENDOR spectra of triplet states often allow—in contrast to doublet states—the direct determination of the signs of hyperfine couplings (see below).

Photosynthetic pigments, i.e., photosynthetic primary donors, antenna Chls and carotenoids have been extensively investigated in their triplet states by transient and pulse ENDOR spectroscopy in order to derive the hfcs of magnetic nuclei like ^1^H and thus the spin density distribution in the triplet state (Di Valentin et al. 1996; Lendzian et al. 1998, 2003; Niklas et al. 2007; Salvadori et al. 2012; Carbonera et al. 2014b; Marchanka et al. 2014). ENDOR spectra have also been reported for many porphyrins, e.g., Kay et al. (1995), Tait et al. (2015), Richert et al. (2017), Barbon et al. (2020) and (bacterio)chlorophyll model systems dissolved in organic solvents or inserted in the protein environment of the Water-Soluble Chlorophyll Protein (WSCP) (Marchanka et al. 2009; Agostini et al. 2017, 2019a, 2020). In the specific case of ^3^P680, the “primary donor” of Photosystem II (PSII), X-band ^1^H-ENDOR spectra have been reported (Di Valentin et al. 1996; Lendzian et al. 2003) while no ENDOR data are available so far for ^3^P700, the primary donor of Photosystem I (PSI).

**Fig. 1:**
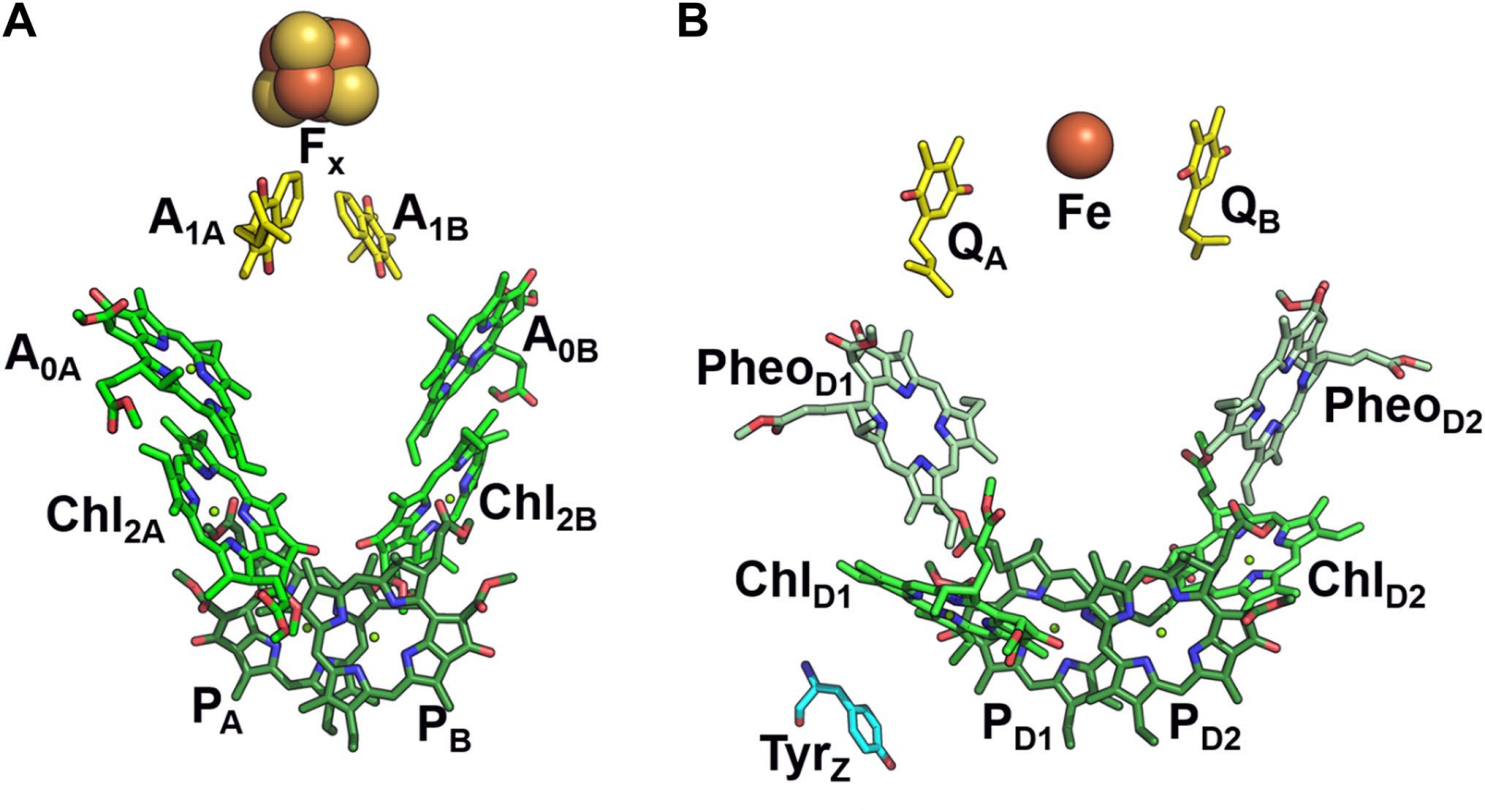
**A** and **B** show the arrangements of electron transfer cofactors in PSI [PDB ID: 1JB0 (Jordan et al. 2001)] and PSII [PDB ID: 3WU2 (Umena et al. 2011)], respectively. Chls constituting the “primary donor” (P_D1_ and P_D2_ for P680, P_A_ and P_B_ for P700) are shown in dark green, other Chls in green, pheophytins (Pheo_D1_ and Pheo_D2_) in pale green, plastoquinones Q_A_ and Q_B_) and phylloquinones A_1A_ and A_1B_) in yellow, tyrosine (Tyr_Z_) in cyan. ET cofactors involved in later steps of charge separation (F_A_, F_B_) have been omitted for clarity, as phytyl and polyisoprene moieties of chlorophylls and quinones, respectively

According to the X-ray crystal structure of PSI from the thermophilic cyanobacterium *Thermosynechocccous (T.) elongatus*, P700 is a Chl (hetero)dimer (see Fig. 1A) which consists of one Chl *a* molecule (P_B_, B-branch) and one Chl *a*′ molecule (P_A_, A-branch) (Jordan et al. 2001; Fromme et al. 2001). Chl *a*′ is the 13^2^ epimer of Chl *a*, in which the two substituents at the position 13^2^ are interchanged. The two Chl rings are approximately parallel to each other and oriented perpendicular to the membrane plane; they partially overlap at the pyrrole rings A and B, with an average interplanar distance of 3.4–3.6 Å. The Mg–Mg distance in this Chl pair is 6.3 Å. Subsequent X-ray and cryo-EM studies have confirmed these results for multiple biological species (Qin et al. 2015; Mazor et al. 2017; Su et al. 2019; Xu et al. 2020; Keable et al. 2021). Several models for the early charge transfer steps in PSI have been discussed; in some charge separation starts from other Chls beside P_A_ and P_B_ (Müller et al. 2003, 2010; Savikhin and Jankowiak 2014; Cherepanov et al. 2020; Gorka et al. 2021). EPR experiments on oriented photosynthetic membranes at low temperature suggested that ^3^P700 is localized on one or more Chl(s) with their plane perpendicular to the membrane (Rutherford and Sétif 1990), which would correspond to P_A_ and/or P_B_ according to the crystal structure. Fourier transform infrared (FTIR) spectroscopy provided evidence that the ^3^P700 triplet is fully localized on P_A_ (Breton 2001, 2006) while optical and magnetic resonance data on a series of PSI mutants were interpreted in terms of triplet exciton localization on P_B_ (Krabben et al. 2000; Witt et al. 2002, 2003). These FTIR and ODMR experiments were done at cryogenic temperatures. At ambient temperature the transient EPR spectra indicate successive delocalization (or hopping) of the triplet exciton over more than one chlorophyll molecule (Sieckmann et al. 1993; Niklas 2007).

The PSII structure (Cardona et al. 2018; Sheng et al. 2019; Umena et al. 2011) shows that the center-to-center (Mg–Mg) distance between the Chls P_D1_ and P_D2_ is about 8.2 Å, further separated from each other than the respective Chls in PSI. The average interplanar distance between the *π*-planes is very similar to PSI, 3.4–3.6 Å (Gorka et al. 2021). In PSII the photochemical trap P680, which absorbs close to 680 nm, is believed to include not only P_D1_ and P_D2_, but also other Chl *a* molecules, e.g., Chl_D1_, Chl_D2_ (see Fig. 1B) (Durrant et al. 1995; Savikhin and Jankowiak 2014; Gorka et al. 2021). Using EPR spectroscopy on oriented photosynthetic membranes (van Mieghem et al. 1991) and PSII single crystals (Kammel et al. 2003), it was shown that the triplet state at low temperatures is located on one of the accessory chlorophylls, Chl_D1_. This localization is in agreement with analysis of optical spectroscopy data (Diner et al. 2001; Zabelin et al. 2016; Takegawa et al. 2019). Despite the long-lived radical cation P680^·+^ and the transient triplet state not being located on the same Chl molecule (Zech et al. 1999; Kawamori et al. 2005), the standard term ^3^P680 for naming the recombination triplet state in PSII is commonly used and will be adopted in the following. At elevated temperatures the transient EPR spectra change, and have been interpreted as delocalization of the triplet exciton over other chlorophyll molecules and possibly a pheophytin (Kamlowski et al. 1996; Frankemöller et al. 1998; Pashenko et al. 2003).

This study gives the most detailed information of the ^1^H hfc tensors of ^3^P680 in PSII and is the first ENDOR study of ^3^P700 in PSI, derived from a direct comparison between the two triplet states using pulse Q-band ^1^H-ENDOR spectroscopy. The assignment of the ENDOR signals is based on comparison with previously reported ENDOR spectra of related systems (Lendzian et al. 2003; Agostini et al. 2017) and density functional theory (DFT) calculations on chlorophyll triplet species (Agostini et al. 2019a). The monomeric nature of both triplet states at cryogenic temperature is confirmed and the changes at elevated temperatures are discussed. Effects of the protein environment on the site of localization of the ^3^Chl are also presented.

## Experimental

### Sample preparation

The *Thermosynechococcus (T.) elongatus* trimeric PSI complexes were a kind gift from P. Fromme, J. Frank and J. Kern (TU Berlin, Germany), and were isolated as previously described (Fromme and Witt 1998). This complex contains all protein subunits and cofactors of Photosystem I, including the core antenna. The micro-crystals obtained were dissolved before EPR samples preparation, ensuring the presence of pure and intact PSI without any contamination of PSII. The sample preparation was performed under dimmed green light.

The protein was concentrated to about 12–15 mM Chl (≈ 0.14 mM RCs). The ^3^P700 EPR samples were prepared by procedures similar to the ones used for generation of the stationary quinone radical anion A_1_^·−^ (Bonnerjea and Evans 1982; Gast et al. 1983; Poluektov et al. 2005) and used the same illumination setup as described previously (Niklas et al. 2009). Sodium dithionite was added to a final concentration of 30 mM in 0.2 M glycine buffer (pH 10). The sample was incubated for 30 min at 4 °C in the dark. Continuous illumination, for reduction of the iron-sulfur centers and the A branch quinone, was done by two 150 W halogen lamps (one from each side) equipped with water filter, cold glass filter and a concentrated CuSO_4_ solution at 240 K for 30 min (photoaccumulation). All steps after obtaining the concentrated PSI solution were performed anaerobically under dimmed green light on ice until illumination was started.

The *Spinacia oleracea* D1D2Cyt*b*_*559*_-complexes (Nanba and Satoh 1987) were a kind gift of A. Holzwarth (MPI for Chemical Energy Conversion, Mülheim/Ruhr, Germany), and were prepared as previously described in van Leeuwen et al. (1991), with the exception that the incubation with Triton X-100 was done three times. The complexes obtained by this method contained six Chl *a*, two pheophytin *a*, and one or two β-carotene molecules. The samples contained no quinones, and most of them have lost the non-heme iron. The complexes were concentrated to an OD_676_ ≈ 200 (≈ 0.3–0.4 mM RCs) using a YM-30 Centricon. The concentrated protein solution was transferred to quartz tubes and quickly frozen in liquid nitrogen. The sample preparation was done under dimmed green light in a cold room.

For all EPR/ENDOR measurements at Q-band, quartz capillaries with an outer diameter of about 2.8 mm and an inner diameter of 2 mm have been used.

### EPR and ENDOR experiments

Q-band pulse EPR and ^1^H-ENDOR experiments were performed on a Bruker ELEXSYS E580-Q spectrometer equipped with a Super Q-FT microwave bridge (Bruker Biospin, Rheinstetten, Germany). A home-built TE_011_-type microwave cavity similar to the one described in (Reijerse et al. 2012) was used, which contains slits to allow in situ light excitation of the sample (Niklas et al. 2009). Light excitation at 532 nm was achieved with the Brilliant Laser system from Quantel. It consists of an OPO, type Vibrant 355 II, pumped by short (≈ 8 ns) light pulses at 355 nm provided by a Nd:YAG Laser. For some measurements, an OPO (GWU model VISIR), pumped by short (≈ 8 ns) light pulses at 355 nm provided by a Nd:YAG Laser system (Spectra Physics, GCR 130) was used. In both setups, the repetition rate was 10 Hz, and the light energy at the cryostat window about 10 mJ per pulse.

Field-sweep echo-detected EPR (FSE-EPR) spectra were recorded using the two-pulse echo sequence (*π*/2–*τ*–*π*–*τ*-echo), where the echo intensity was registered as a function of the magnetic field. Microwave (MW) pulses of *π*/2 = 40 ns, *π* = 80 ns and *τ* = 300–400 ns were used. All pulse EPR spectra were corrected for the ‘dark’ background (if present) recorded 20 ms after the Laser flash.

^1^H-ENDOR on ^3^P680 was recorded using the Davies ENDOR sequence (*π*–*t*–*π*/2–*τ*–*π*–*τ*-echo) (Davies 1974) with an inversion pulse *π* = 200 ns, *t* = 20 μs, radiofrequency (RF) *π*-pulse of 16–17 μs and a detection sequence similar to the FSE-EPR experiment. The sequence of MW and RF pulses and the detection was repeated and a stationary background spectrum (recorded 20 ms after the Laser flash) subtracted (if present). The generation of RF pulses and the signal acquisition was done by an external PC equipped with the SpecMan program (Epel et al. 2005) and an SMT 02 Rhode and Schwarz synthesizer and a high-speed digitizer (Acqiris AP235). An ENI 3200L 300 W RF amplifier was used for these measurements.

^1^H-ENDOR on ^3^P700 was recorded under conditions similar to ^3^P680, but a 2.5 kW AR2500L RF amplifier (Amplifier Research) was used, which allowed shorter RF pulses (down to 7 µs) and thus a shorter time for the pulse sequence which increased the S/N ratio.

## Results

In this section we will first briefly introduce the principles of EPR and ENDOR performed on spin-polarized chlorophyll triplet states to provide a better understanding of the following experiments and their analyses. It is important to note that in contrast to a ^3^Chl in solution or in antennas, which is usually formed via ISC, the triplet states in the reaction centers PSI and PSII are derived from a RP state formed in the charge separation process (Okamura et al. 1987; Telfer et al. 1988; Setif and Bottin 1989; Budil and Thurnauer 1991; Brettel 1997; Lubitz 2002). The initially formed singlet RP can form a triplet RP by action of different magnetic interactions in the two radicals; a recombination of the triplet RP then leads to a Chl triplet state in the photosystem (^3^P).

### EPR and ENDOR on triplet states in oxygenic photosynthesis

**Fig. 2:**
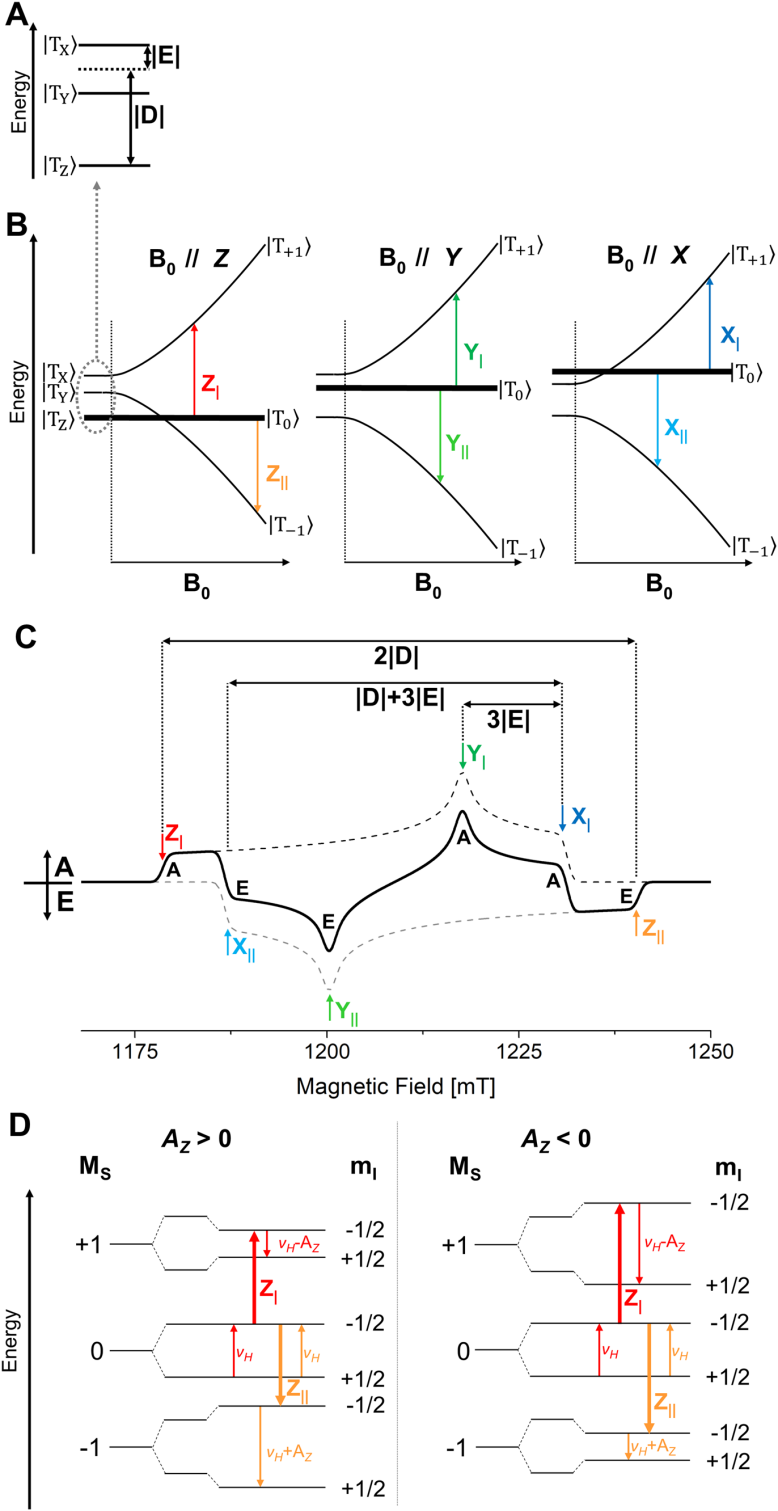
Triplet (*S* = 1) spin energy levels of ^3^Chl in zero and high magnetic field, spin polarized EPR spectrum, and coupling to nuclear spins: **A** Triplet spin energy levels at zero field (enlarged). D and E are the ZFS parameters; for ^3^Chl D > 0, E < 0. **B** Triplet energy levels in a high external magnetic field. The thickness of the lines indicates the population of the respective level. Here the |*T*_0_ > level is selectively populated due to the radical pair (RP) mechanism, and the triplet is an ST_0_ triplet. The other levels (|*T*_+1_ > and |*T*_−1_ >) are initially not populated. The colored arrows indicate the allowed Δ*M*_S_ = 1 EPR transitions for ZFS axes parallel to the magnetic field, three in absorption, (Z_|_, Y_|_, X_|_) and three in emission (Z_||_, Y_||_, X_||_). Note that the electron spin and ZFS energies are not to scale; electron Zeeman interactions at Q-band are much larger than the ZFS parameters D and E for ^3^Chl (*ν*_e_ ≈ 34 GHz ≫|D|≈ 850 MHz, |E|≈120 MHz). See Fig. S1 for a to scale depiction. **C** Spin polarized transient EPR spectrum corresponding to scheme (B). The absorptive and the emissive spectra are indicated by dashed lines and the sum spectrum by a solid black line; A = absorption, E = emission. The elucidation of the ZFS parameters |D| and |E| is indicated. **D** Scheme showing the EPR and ENDOR transitions of a single proton ^1^H (*I* = ½) coupled to the triplet state (*S* = 1) for two specific magnetic fields corresponding to the spectral positions Z_|_ and Z_||_ in the triplet EPR spectrum for a positive and a negative hyperfine coupling constant A_Z_. Note that the electron spin and nuclear spin energies are not to scale; electron Zeeman interactions are about 660 times larger than nuclear Zeeman interactions of ^1^H (*ν*_e_ ≈ 34 GHz ≫ *ν*_N_(^1^H) ≈ 52 MHz), which are larger than ^1^H hyperfine couplings in ^3^Chl at Q-band (|*A*|< 20 MHz)

Figure 2 shows the Zero Field Splitting (ZFS) of a triplet state (*S* = 1) in absence of an external magnetic field (A) and the splitting of the three spin energy levels at high field (B) for the three canonical orientations X, Y, and Z of the triplet. The ZFS parameter is positive, D > 0, as expected for *π*–π* triplet states of porphyrin derivatives like chlorophylls (Budil and Thurnauer 1991; Richert et al. 2017). The sublevel with *M*_S_ = 0 is exclusively populated at high magnetic field, as expected for triplet states formed by RP recombination (ST_0_ triplet). Due to selective population of this triplet sublevel a strong polarization is obtained in the time-resolved EPR experiment (Angerhofer 1991). For such triplet states, the spin polarization in the Chl triplet state EPR spectrum is expected to be AEEAAE (A = absorption, E = emission). An illustrative example is shown in (C). This spin polarized spectrum can be considered as the sum of two powder spectra (dashed lines), one from the absorptive transitions between *M*_S_ = 0 and *M*_S_ =  + 1 spin energy levels (Z_|_,Y_|_, X_|_) and one from the emissive transitions between *M*_S_ = 0 and *M*_S_ = − 1 spin energy levels (Z_||_, Y_||_, X_||_). Since the absorptive and the emissive spectrum are shifted with respect to each other as a consequence of the spin–spin dipolar interaction between the two unpaired electrons, the sum spectrum shows a characteristic polarization pattern which cannot be created by ISC (Budil and Thurnauer 1991; Lubitz 2002; Lubitz et al. 2002; Richert et al. 2017).

In Fig. 2D the spin energy levels obtained from the hyperfine interaction of the triplet spin (*S* = 1) with one nuclear spin (*I* = ½) in the high-field limit are shown. The EPR transitions are indicated by thick arrows, the ENDOR transitions by thin arrows. In a doublet state (*S* = ½) the ENDOR transition frequencies are ^2^*ν*_ENDOR_ = |*ν*_H_ ± A/2| and are thus symmetrically spaced around the nuclear Zeeman frequency *ν*_H_ if half the hyperfine coupling is smaller than the Larmor frequency (|A|/2 < *ν*_H_). In contrast, in a triplet state, a strong and narrow line is expected from the *M*_S_ = 0 manifold at the Larmor frequency *ν*_H_, and further ENDOR transitions occur either at higher or lower frequencies with respect to *ν*_H_ depending on the sign of the hyperfine interaction tensor element (here A_Z_) if the hyperfine coupling is smaller than the Larmor frequency (|A|< *ν*_H_). As an example, we consider the triplet spin energy levels for the Z orientation, including first-order hyperfine interaction A_Z_ with one proton. For each EPR transition, there are two ENDOR resonance frequencies according to the triplet ENDOR resonance condition (Lubitz 2002):

1 $${}^{3}\nu_{{{\text{ENDOR}}}} = \, |\nu_{{\text{N}}} - {\text{ M}}_{{\text{s}}} {\text{A}}|$$

It follows that those ENDOR transitions which do not stem from a nuclear transition in the *M*_S_ = 0 manifold are either at the higher or lower frequency side with respect to the central *ν*_H_ transition, depending on the specific canonical transition. For the absorptive Z_|_ transition (*M*_S_ = 0 to *M*_S_ =  + 1) of a triplet state with *D* > 0, the ENDOR lines occur on the low (if A_Z_ > 0) or high (if A_Z_ < 0) frequency side with respect to the Larmor frequency *ν*_H_. The opposite situation is encountered for the Z_||_ transition. Therefore, if the sign of the ZFS parameter D is known, the sign of the hfcs can be directly derived from the spectral position corresponding to the lines at higher/lower frequencies with respect to *ν*_H_. If the sign of a hyperfine coupling is known (*e.g.,* positive for methyl group protons), the sign of D can immediately be inferred. Moreover, in principle it is sufficient to collect the ENDOR spectra at just half of the field positions corresponding to the EPR spectrum turning points, one for each canonical orientation. This last statement holds only if all ENDOR lines are clearly visible, which is not always the case (e.g., ENDOR transitions at low RF frequencies have often low intensity), and no other magnetic nuclei like ^14^N contribute to the same spectral range of the ENDOR spectra; for the systems under study here (^3^Chl) at Q-band (34 GHz, ≈ 1.2 T, *ν*_N_(^1^H) ≈ 52 MHz) this is the case, but at lower frequencies/fields this may be different.

#### Orientation selection ENDOR and hfc assignments

**Fig. 3:**
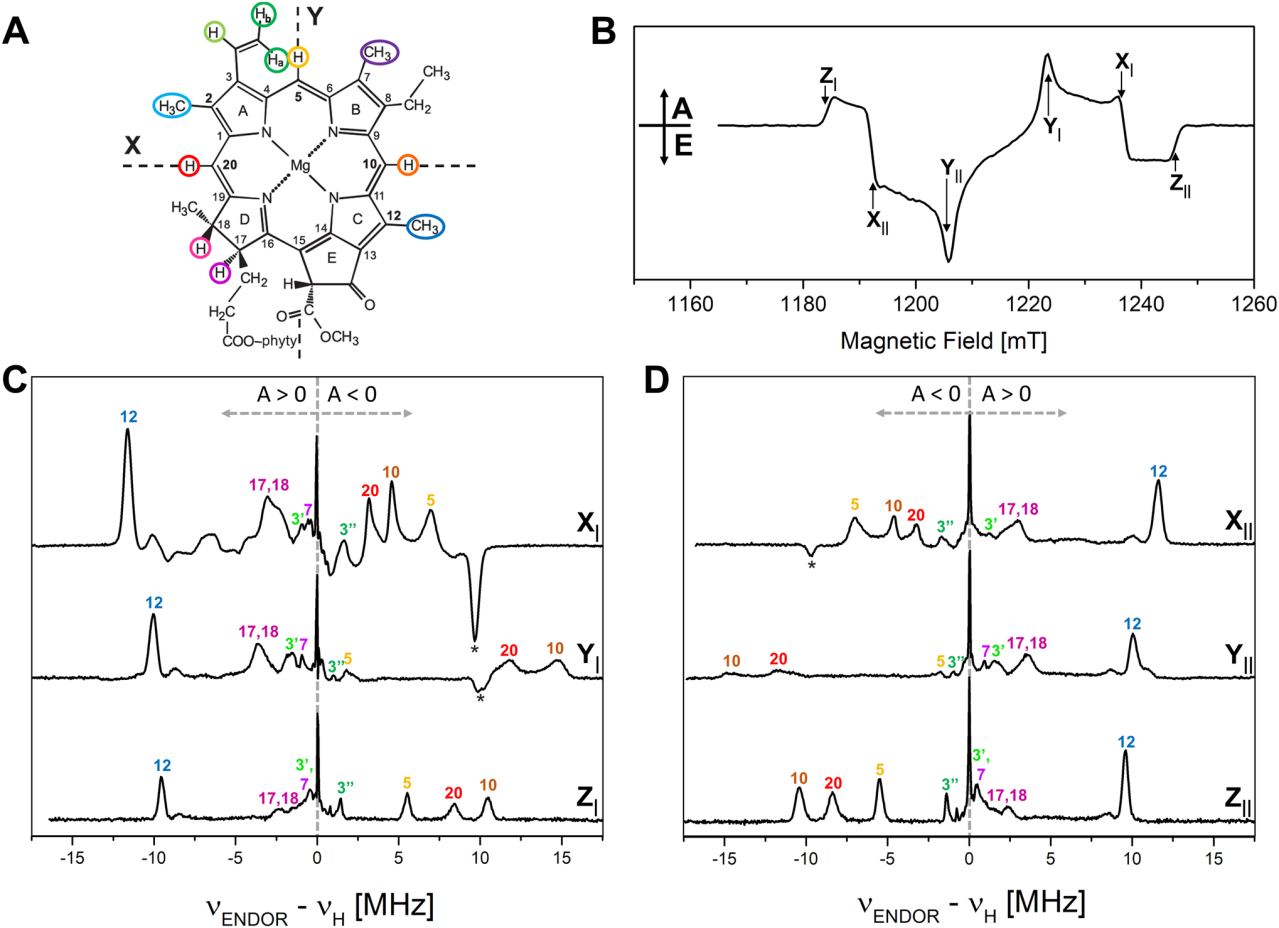
Q-band pulse EPR and Davies ^1^H-ENDOR spectra of ^3^P680 in D1D2Cyt*b559* particles at 10 K; 1 μs DAF. **A** Scheme of the Chl *a* structure with the orientation of the ZFS axes X, Y (the axis Z is perpendicular to the molecular plane). The α (directly bound to the *π*-system: methine **5**, **10**, **20**; vinyl group **3′**, **3″**) and β protons (methyl **2**, **7**, **12** and positions **17**, **18**) are highlighted by colored circles (only for |A_iso_|> 1 MHz) (Agostini et al. 2019a)). **B** Pulse FSE Q-band EPR spectrum (ZFS canonical orientations are labeled). The ENDOR spectra **(C** and **D)** have been recorded at fields corresponding to the canonical orientations of ^3^P680 (X_|_ at 1237 mT; Y_|_ at 1224 mT; *Z*_|_ at 1185 mT; X_||_ at 1193 mT; Y_||_ at 1206 mT; Z_||_ at 1246 mT). The frequency scale gives the deviation from *ν*_H_ for better comparison, since the differences in magnetic fields cause significant changes of the proton Larmor frequency. All ENDOR spectra are presented as absorptive spectra. Prominent ENDOR lines of opposite polarization are marked with asterisks. The experimental details are given in the “Experimental” section. The numbers used for assignments of lines refer to the IUPAC numbering of the Chl *a* structure, shown in panel **A**. Further explanations of the principles of ENDOR on ^3^P680 are provided in Fig. S4

In Fig. 3A the Chl *a* structure including the ZFS tensor axes X, Y, Z for the triplet state are given that have been derived for ^3^Chl *a* in (Vrieze and Hoff 1995; Lendzian et al. 2003). ENDOR experiments performed with the magnetic field in the EPR positioned at Z_|_ or Z_||_ are selecting molecules oriented with their molecular (*π*) plane perpendicular to *B*_0_, leading to a strong selection of nuclear transitions along the Z axis in the ENDOR spectrum and result in single crystal-like ENDOR spectra (Hoffman et al. 1993). For protons located in the plane of the *π*-system this is in very good approximation the A_Z_ component of the hfc tensor. This includes the methine α-protons and also the β-protons of freely rotating methyl groups. ENDOR experiments along the other two ZFS tensor axes X and Y select other components. While at X orientation also a single crystal-like ENDOR spectrum can be obtained, at Y orientation molecules with a variety of orientations with respect to the magnetic field will be excited (Lendzian et al. 2003; Richert et al. 2017). A further complication of the ENDOR spectra obtained at X and Y orientation is that also the other EPR transition is excited (see Figs. 2C and S4), which results in an ENDOR spectrum which is the overlap of an emissive and absorptive ENDOR spectrum, which reduces the intensity and complicates the ENDOR spectrum.

The hfcs measured along X and Y could again be very close to the principal values of the hfc tensor in favorable cases as for the methine protons at positions **5**, **10**, and **20** and approximately also for the rotating methyl group protons at positions **12** and **2** (Fig. 3A). This acquisition of ENDOR spectra at all canonical orientations often allows, even for randomly oriented molecules in frozen solution, a full determination of the principal hfc tensor elements of a particular nucleus. The possibility to directly obtain the signs of the hfc values from the ENDOR spectrum is also very helpful for assignment purposes. For example, it is known that in Chls the methine α-protons show large negative hfcs, whereas the methyl group protons exhibit positive hyperfine couplings. The magnitude of particular hfcs in the triplet state can be estimated as the mean of the respective values in the radical cation and radical anion as demonstrated earlier (Carrington and McLachlan 1969; Lendzian et al. 1998). This approach relies on the simple assumption that in the triplet state one unpaired electron is delocalized in the HOMO and one in the LUMO of the molecule and interact with the various nuclei. This enables a rough experimental estimate of the spin density distribution and the hfcs of the triplet state. It requires that the data for the related radical ions are known from ENDOR experiments, which is the case for the chlorophyll radicals (Lubitz 1991). The hfcs of the triplet can then be verified by quantum chemical calculations.

### EPR and ENDOR of ^3^P680 in PSII

The pulse Q-band Field-Swept Echo detected (FSE)-EPR spectrum of photo-induced ^3^P680, in a frozen solution of a PSII preparation (D1D2Cyt*b*_*559*_) at 10 K is shown in Fig. 3B. These D1D2Cyt*b*_*559*_ complexes have been selected since they are thought to present the inner RC of PSII in a small compact form lacking the water oxidation unit and the quinone acceptors. This makes them ideal for studying the primary steps of light-induced charge separation and triplet formation without requiring biochemical treatment with reducing agents and light. The spectrum clearly shows the polarization pattern AEEAAE predicted for a ST_0_ triplet with D > 0. The ZFS parameters obtained from the simulation of the spectra (|D|= 0.0288 cm^−1^, |E|= 0.0043 cm^−1^) are, within error, identical to those obtained previously by pulse and transient EPR at X-band (9–10 GHz) and D-band (130 GHz) (Di Valentin et al. 1996; Lendzian et al. 2003; Pashenko et al. 2003) and are very similar to those of monomeric ^3^Chl *a *in vitro (Di Valentin et al. 1996, 2009; Poluektov et al. 2002; Lendzian et al. 2003)*,* see Table 1. In contrast to X-band EPR, the highly isotropic g-tensor of ^3^P680 is resolved at Q-band and found to be essential axial (*g*_*X*_, *g*_*Y*_ > *g*_*Z*_). The principal *g-*values of ^3^P680 are in good agreement with those determined in a D-band study (Pashenko et al. 2003). The excellent agreement of ZFS parameters obtained over a wide frequency range (9–130 GHz) shows that the g-tensor and ZFS-tensor axes are in good approximation collinear. The ZFS parameters and g tensor values obtained from simulations of transient EPR spectra at X- and Q-band (Fig. S2) are given in Table 1. The pulse EPR spectra typically have reduced signal intensities as compared to transient (direct detection) EPR spectra for non-canonical orientations (Lendzian et al. 2003). An additional difference is the presence of nuclear modulation (ESEEM) in the pulse spectra; in particular the Chl macrocycle nitrogen atoms can cause significant changes in electron spin echo intensities depending on details of the pulse sequence (Schweiger and Jeschke 2001). In addition to the echo-detected pulse spectra, FID-detected pulse spectra have also been recorded, which are more similar to the transient EPR spectra, demonstrating that nuclear modulation effects are indeed one contributing factor to the differences between echo-detected pulse EPR spectra and transient EPR spectra (Figs. S2 and S3). The similarity of both ZFS parameter |D| and |E| in ^3^P680 and monomeric ^3^Chl in solution has been interpreted as a localization of the triplet exciton on a monomeric Chl in PSII. However, the hyperfine coupling constants are more sensitive probes of the exciton delocalization, as discussed below. We have recorded EPR spectra at longer delay after flash (DAF) times and DAF-decays to confirm that at 10 K no substantial relaxation appears in the time required to perform the pulse sequence (not shown), which is in agreement with previous work at X-band (Lendzian et al. 2003).

**Table 1:** Experimental ZFS parameters D and E and principal values of the g-tensors for ^3^P680 and ^3^P700 compared with ^3^Chl *a* (cryogenic temperatures only; set of selected references)

Triplet state	Species/solvent	Preparation	ZFS Parameter |D|	ZFS Parameter |E|	*g* values^a^	References
^3^Chl *a*	90:10 toluene:pyridine		284 ± 1 × 10^−4^ cm^−1^	41.3 ± 0.2 × 10^−4^ cm^−1^	*g*_*X*_ = 2.00344( ±)0.00009, *g*_*Y*_ = 2.00382( ±)0.00009, *g*_*Z*_ = 2.00265( ±)0.00009	Poluektov et al. (2002)
^3^Chl *a*	Polymethylmethacrylate		306 ± 1 × 10^−4^ cm^−1^	43 × 10^−4^ cm^−1^		Di Valentin et al. (1996)
^3^Chl *a*	2-Methyltetrahydrofuran (MTHF)		282 × 10^−4^ cm^−1^ ± 1%	38 × 10^−4^ cm^−1^ ± 8%		Lendzian et al. (2003)
^3^P680	*Spinacia oleracea* (spinach)	D1D2Cyt*b*_559_	288 ± 2 × 10^−4^ cm^−1^	43 ± 2 × 10^−4^ cm^−1^	*g*_*X*_ = 2.0031( ±)0.0002, *g*_*Y*_ = 2.0032( ±)0.0002, *g*_*Z*_ = 2.0022( ±)0.0002	This work
^3^P680	*Spinacia oleracea* (spinach)	D1D2Cyt*b*_559_	30.9 ± 0.2 mT ≈289 ± 2 × 10^−4^ cm^−1^	4.6 ± 0.1 mT ≈43 ± 1 × 10^−4^ cm^−1^	*g*_*X*_ = 2.00324( ±)0.00004, *g*_*Y*_ = 2.00306( ±)0.00004, *g*_*Z*_ = 2.00231( ±)0.00004	Pashenko et al. (2003)
^3^P680	*Pisum sativum* (pea)	D1D2Cyt*b*_559_	287 × 10^−4^ cm^−1^ ± 1%	43 × 10^−4^ cm^−1^ ± 8%		Lendzian et al. (2003)
^3^P680	*Spinacia oleracea* (spinach)	D1D2Cyt*b*_559_	287 ± 1 × 10^−4^ cm^−1^	42 × 10^−4^ cm^−1^		Di Valentin et al. (1996)
^3^P680	*Spinacia oleracea* (spinach)	Core complexes (Q_A_^2−^)	286 ± 1 × 10^−4^ cm^−1^	44 × 10^−4^ cm^−1^		Feikema et al. (2005)
^3^P680	*Spinacia oleracea* (spinach)	Thylakoids	285 × 10^−4^ cm^−1^	45 × 10^−4^ cm^−1^		Santabarbara et al. (2002)
^3^P680	*Chlamydomonas reinhardtii*	Thylakoids	285 × 10^−4^ cm^−1^	45 × 10^−4^ cm^−1^		Santabarbara et al. (2007)
^3^P700	*Thermosynechococcus elgongatus*	PSI trimer	278 ± 2 × 10^−4^ cm^−1^	38 ± 2 × 10^−4^ cm^−1^	*g*_*X*_ = 2.0033( ±)0.0002, *g*_*Y*_ = 2.0030( ±)0.0002, *g*_*Z*_ = 2.0021( ±)0.0002	This work
^3^P700	*Synechococcus lividus* (deuterated)	PSI trimer	280 ± 1 × 10^−4^ cm^−1^	39.0 ± 0.2 × 10^−4^ cm^−1^	*g*_*X*_ = 2.00369( ±)0.00009, *g*_*Y*_ = 2.00323( ±)0.00009, *g*_*Z*_ = 2.00252( ±)0.00009	Poluektov et al. (2002)
^3^P700	*Synechococcus* sp.	PSI complexes	289 ± 15 × 10^−4^ cm^−1^	39.0 ± 2 × 10^−4^ cm^−1^		Sieckmann et al. (1993)
^3^P700	*Spinacia oleracea* (spinach)	CP1 particles	835–845 MHz ≈ 279–282 × 10^−4^ cm^−1^	113–117 MHz ≈38–39 × 10^−4^ cm^−1^		Vrieze et al. (1996)
^3^P700	*Spinacia oleracea* (spinach)	Thylakoids	277–281 × 10^−4^ cm^−1^	36–37 × 10^−4^ cm^−1^		Santabarbara et al. (2002)
^3^P700	*Chlamydomonas reinhardtii*	Thylakoids	277–281 × 10^−4^ cm^−1^	38–39 × 10^−4^ cm^−1^		Santabarbara et al. (2007)

^a^The g-tensor and ZFS-tensor principal axes are taken as collinear. Absolute errors in g-values are typically larger than the relative errors given

Pulse ENDOR experiments on ^3^P680 in the D1D2Cyt*b*_*559*_ particles have been performed with field settings corresponding to all the canonical ZFS EPR transitions. This allows the selective excitation of molecules with the ZFS tensor axes (X, Y, or Z) parallel to the magnetic field, yielding single crystal-like ENDOR spectra for Z_|_ and Z_||_ and further orientational information for the other axes, from which a complete set of A_ii_ hfc tensor components of α- and β-protons (including signs) in the reference frame of the ZFS tensor can be extracted. Note, that at X orientation also a single crystal-like ENDOR spectrum can be obtained, but ENDOR signals stemming from the *M*_S_ state with opposite signs overlap and have the opposite polarization (absorptive vs emissive). The signs, the absolute values and the orientations of the hyperfine tensor components are all important for the assignment to specific protons and the consequent mapping of the spin-density distribution of the unpaired triplet electrons over the conjugated Chl macrocycle. From theoretical considerations, freely rotating methyl groups are expected to show positive hfcs with almost axial symmetry, an anisotropy less than 10% of the respective isotropic hfcs and the major hfc value along the C(*π*)–CH_3_ bond axis, while α-protons are characterized by hfcs with much larger anisotropies, negative signs, and a different orientation with respect to the C(*π*)–H bond. These relations have been well established for a wide variety of organic radicals (Carrington and McLachlan 1969; Gordy 1980).

Pulse Q-band ^1^H-ENDOR spectra of ^3^P680 at 10 K, recorded at all the canonical field positions, are presented in Fig. 3C and D. The frequency scale in this figure measures the deviations from the proton Larmor frequency and the hfcs correspond to the frequency shift between the ENDOR line and *ν*_H_ according to the triplet ENDOR resonance condition (see Eq. 1). The ENDOR transitions are labeled as positive or negative with respect to their appearance in the spectrum relative to *ν*_H_, considering the positive sign of D for the Chl triplet state. Note that the emissive ENDOR spectra have been inverted to facilitate comparison.

All the ENDOR spectra show, in addition to the narrow free proton line at the proton Larmor frequency, signals from protons with positive and negative hfcs. As expected, in Fig. 2C and Fig. 2D the positions of the ENDOR lines with respect to *ν*_H_ are exchanged, when exciting the corresponding low-field or high-field canonical EPR transition (easiest to see for Z canonical transitions Z_|_ and Z_||_). The ENDOR spectra are rich in structure and the presence of narrow lines in the spectra indicates that single-crystal-like positions have been selected for Z and also X, and even at Y position highly resolved ENDOR spectra are obtained. Indeed, the ENDOR linewidths of some peaks from weak to medium hfcs were found to be smaller than 100 kHz. This is already at the limit of the resolution in the ENDOR spectra. Hence, additional high resolution ENDOR spectra with more points and longer RF pulses were recorded. They did not show significantly improved resolution (data not shown). Additional lines, some prominent ones marked with an asterisk, were observed in the ENDOR spectra at the X and Y canonical orientations showing opposite polarization. They derive from contributions from non-canonical orientations of the overlapping other electron spin transition (Figs. 2 and S4). These ENDOR lines with inverted polarization are expected to be most pronounced for protons with highly isotropic hfc tensors, like protons of the rotating methyl group **12** (see “Discussion” section).

There have been two previous ^1^H ENDOR studies on ^3^P680 in D1D2Cyt*b*_*559*_ complexes; a transient ENDOR study at X-band (Di Valentin et al. 1996), and a pulse (Davies) ENDOR study at X-band (Lendzian et al. 2003). The hfcs determined in those works are in excellent agreement with the ones determined here (Di Valentin et al*.* reported only the A_Z_ component of the hyperfine tensors). Pulse (Davies) ENDOR is usually considered to be better than transient ENDOR for the detection of strongly coupled protons, and less suited for the detection of weak to medium coupled protons (where transient ENDOR is better). However, in our pulse Q-band ENDOR spectra more lines from weakly coupled protons are resolved than in the previous X-band transient- and X-band pulse- ENDOR studies. We attribute the higher resolution achieved here to a combination of longer RF and MW pulses (200 ns inversion *π*-pulse) and higher magnetic field (1.2 T vs 0.35 T), where off-diagonal elements of the hyperfine tensors **A** can be neglected. In the case of ^3^Chl *a* in WSCP, Mims ENDOR spectra were also recorded (Agostini et al. 2017, 2020), which are characterized by an intrinsically higher resolution of small hfcs but suffer from blind spots (Gemperle and Schweiger 1991). The corresponding hyperfine structure in the proximity of the free proton line resembles the one for ^3^P680 at Q-band.

The ZFS and hyperfine tensor axes for the three methine α-protons and for the β-protons of methyl **12** can be considered collinear in good approximation. Simulations show that for the anisotropic methine α-protons an in-plane rotation of only 20° already leads to pronounced changes in the simulated ENDOR spectra recorded at X orientation (see Fig. S5), which is not in agreement with our experimental observations. For the more isotropic methyl group protons the effects are less obvious (see Fig. S6). On the basis of an approximate collinearity between the ZFS and the hfc tensor principal axes (see Fig. 3A; methine protons **10**, **20** and **5** are collinear to X, X and Y, respectively; the methyl **12** is approximately collinear to X), the hyperfine components measured in the ZFS frame may be taken as the principal components of the proton hyperfine tensors correctly considering the respective orientations of α- and β-protons within the Chl structure/molecule. From the trace of the principal components the isotropic hyperfine constants can be derived. The magnitude and signs of the ^1^H hfcs and their tentative assignments are presented in Table 2. These assignments are based on (i) the well-known orientation dependence of hyperfine couplings of protons directly connected (α-protons) to, or one bond away (β-protons) from the *π*-system (McConnell et al. 1960; Heller and McConnell 1960; Carrington and McLachlan 1969; Gordy 1980), (ii) the previous ENDOR studies on ^3^P680 (Di Valentin et al. 1996; Lendzian et al. 2003), and (iii) DFT calculations on ^3^Chl *a* (Agostini et al. 2019a). A comparison with hfcs derived for ^3^Chl *a *in vitro (Lendzian et al. 2003) and in WSCP (Agostini et al. 2017) is also reported in Table 2. The most important hfcs and their assignments are discussed in the following.

(i) In the region of the ENDOR spectra where positive hfcs are detected, a prominent strong narrow line is distinguishable at each orientation selected by the field position (largest positive hfc), which can be attributed to protons belonging to a freely rotating methyl group, on the basis of the sign of the hfc and of the small anisotropy. This line is also present in all previous ENDOR spectra of ^3^P680 and of ^3^Chl *a* both in vitro and in the WSCP protein matrix. The largest hyperfine coupling is visible in the ENDOR spectrum recorded at X orientation, and the largest component of a methyl group hfc tensor is along the C-CH_3_ bond, which narrows the assignment of this line to methyl groups **2** or **12**. The estimate derived from the magnitude of this coupling in the Chl radical cation and anion clearly assigns this hfc to methyl **12** (Lubitz 1991)**,** which is further corroborated by DFT calculations. This assignment is in agreement with the two previous ENDOR studies of ^3^P680.
(ii) The second largest positive hfc (taken from the weak line next to the methyl **12**) in the spectrum could arise from the methyl group at position **2**, but this coupling is much larger both than the corresponding hfc found for ^3^P700 (see below), and the corresponding values reported for ^3^Chl *a *in vitro and in WSCP. Also, its intensity is much lower than that of the line assigned to methyl **12**. This weak line was already observed in the ENDOR spectra of ^3^P680 at X-band and could be from a proton of a non-rotating methyl group or another β-proton which has a fixed angle with respect to the Chl *π*-system. In a follow-up study, we have obtained ENDOR spectra of ^3^P680 from another, larger and more complex PSII preparation (monomeric PSII core complexes from *T. elongatus* (Nowaczyk et al. 2006)) and compared them with those from the D1D2Cyt*b*_*559*_ spinach preparation. Additional ENDOR lines could be clearly seen that belong to a methyl group with the right characteristics and the right magnitude for the “missing signal” of methyl group **2**, see Table 2 (S. Prakash, J. Niklas, and W. Lubitz, manuscript in preparation). All other hfcs of ^3^P680 in the PSII core complexes are very close to those obtained for ^3^P680 in the D1D2Cyt*b*_*559*_ complex (within ± 0.2 MHz).
(iii) The other positive hfcs (broader contributions), which were not detected in the ENDOR spectra of ^3^P680 at X-band, are assigned to the β-protons **17** and **18**, but they cannot be distinguished from each other. Among the additional lines present in the vicinity of the free proton line, it is also possible to detect and tentatively assign the contribution of another methyl group (position **7**). Thus, all larger contributions from methyl protons and other β-protons have been detected for ^3^P680.
(iv) In the region of the ENDOR spectra, where negative hfcs are detected belonging to α protons, three signals are present which can be assigned to methine protons **5**, **10** and **20**, due to their sign, large anisotropy and relative magnitude at the different canonical orientations, in agreement with DFT calculation. These lines were already present in the pulse X-band ENDOR spectra of ^3^P680 (Lendzian et al. 2003) and the assignment is now confirmed.
(v) A tentative assignment of the lines belonging to vinyl protons **3ʹ** and **3ʹʹ** is also given in Table 2 to complete mapping of the small hfcs (hfcs < 1 MHz have not been further considered). All these assignments are based on experimental evidence and supported by earlier DFT calculations on ^3^Chl *a* reported in Table 2 (Agostini et al. 2019a).

**Table 2:**
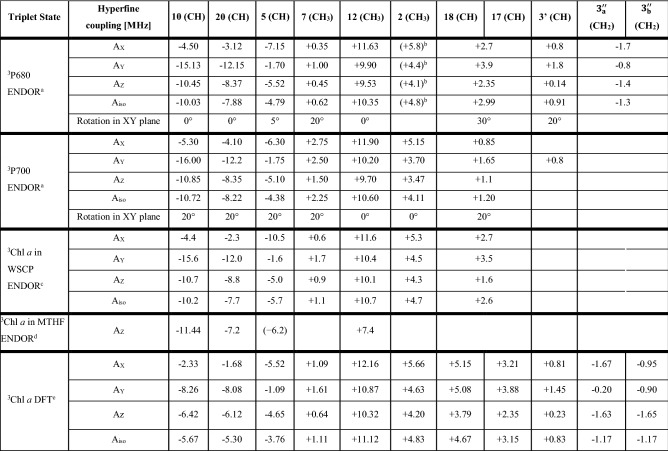
Experimental and calculated ^1^H hfcs of ^3^P680, ^3^P700 and other ^3^Chl *a* species, comparison with DFT calculations

**Fig. 4:**
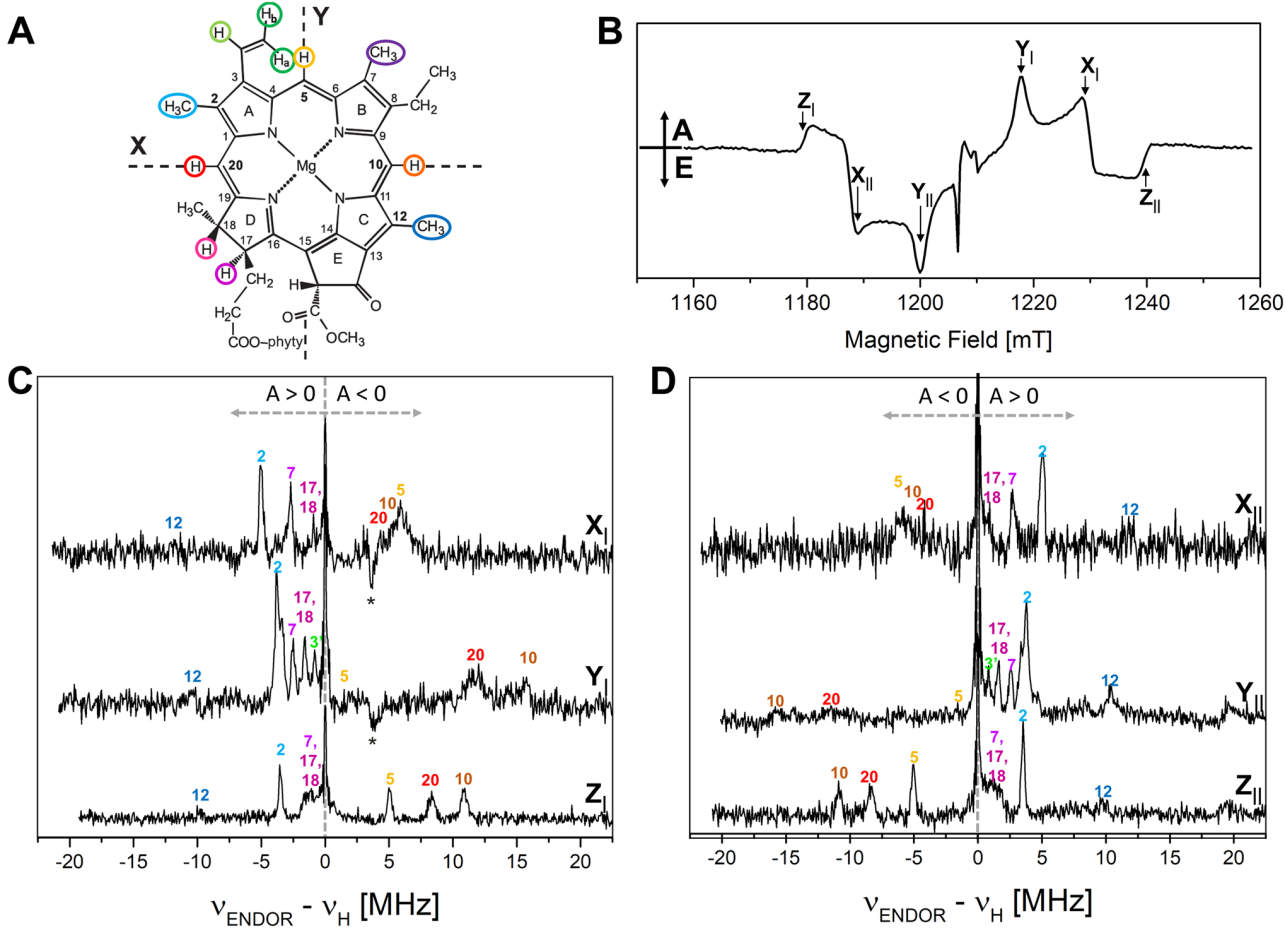
Q-band pulse EPR and Davies ^1^H-ENDOR spectra of ^3^P700 at 30 K; 1 μs DAF. **A** Scheme of the Chl *a* structure with the orientation of the ZFS axes X and Y (the axis Z is perpendicular to the molecular plane). The α (directly bound to the *π*-system: methine **5**, **10**, **20**; vinyl group **3′**, **3″**) and β protons (methyl **2**, **7**, **12** and positions **17**, **18**) are highlighted by colored circles (only for |A_iso_|> 1 MHz) (Agostini et al. 2019a)). **B** Pulse Q-band EPR spectrum (the ZFS canonical orientations are labeled; the strong signals at around *g* = 2 are due to the radical pair P700^·+^A_1_^·−^ and the radicals A_0_^·−^/A_1_^·−^ whose signals could not completely be removed by subtraction of the dark background due to saturation of the detection system, see text). The ENDOR spectra (**C** and **D**) have been recorded at fields corresponding to the canonical orientations of the ^3^P700 marked in the pulse EPR spectrum (X_|_ at 1229 mT; Y_|_ at 1218 mT; Z_|_ at 1180 mT; X_||_ at 1189 mT; Y_||_ at 1200 mT; Z_||_ at 1239 mT). The frequency scale gives the deviation from *ν*_H_. For better comparison, all ENDOR spectra are presented as absorptive spectra; ENDOR lines of opposite polarization are marked with asterisks. The experimental details are given in the “Experimental” section. The assignment labels refer to the IUPAC numbering of the Chl *a* structure, reported in (**A**). Further explanations of the principles of ENDOR on ^3^P700 are provided in Fig. S4

Displayed are α and β protons (for β protons, only those with |*A*_iso_|> 1 MHz). The ^3^P680 and ^3^P700 experimental values were derived from simulations of the Davies ^1^H-ENDOR spectra (Figs. 3 and 4). Simulations are reported in the SI (Figs. S7 and S9). WSCP and in vitro experimental values and the DFT calculated values are derived from previous investigations (Lendzian et al. 2003; Agostini et al. 2017, 2019a)

^a^The X, Y, Z, subscripts of the hfc components are referred to the ZFS reference frame

^b^Hfcs for methyl group **2** in ^3^P680 are taken from monomeric PSII core complexes with doubly reduced Q_A_ (S. Prakash, J. Niklas, and W. Lubitz, manuscript in preparation)

^c^Previously published in Agostini et al. (2017)

^d^Previously published in Lendzian et al. (2003)

^e^The hfc tensors have been previously calculated in Agostini et al. (2019a), level of theory: COSMO-BP86/QZ4P//BP86/TZ2P

### EPR and ENDOR of ^3^P700 in PSI

The measurements conducted on ^3^P700 turned out to be more demanding than those on ^3^P680, which is probably the reason that so far no ENDOR study of ^3^P700 has been performed. A major difference is the size of the protein under investigation: PSI is a huge protein complex (monomer mass in cyanobacteria is ≈356 kD) while the D1D2Cyt*b*_*559*_ complex is much smaller and higher protein concentrations can be achieved. The significantly larger number of Chls per RC in PSI (≈ 16 times the Chls per D1D2Cyt*b*_*559*_ complex) makes the sample optically denser and thus more difficult to excite all RCs with the Laser pulse. However, this is partially compensated for since PSI has a large intrinsic antenna funneling the light energy to the RC. In addition, the necessary pre-reduction with sodium dithionite and the photoaccumulation procedure (see “Experimental” section) likely also causes a somewhat lower triplet yield, since some fraction of RCs will not yet have quinone A_1_ reduced, while another fraction of RCs already has chlorophyll A_0_ reduced (Poluektov et al. 2005). Both of these fractions do not contribute to the ^3^P700 signal. Furthermore, even at low temperatures photochemical side reactions are not completely suppressed. The main problem is the accumulation of a stationary background signal assigned to the radical anion of the Chl acceptor (A_0_^·−^) under the repetitive light excitation of the samples (Laser operating at 10 Hz). As mentioned above, PSI molecules with photoaccumulated A_0_^·−^ do not contribute anymore to the ^3^P700 signal (Bonnerjea and Evans 1982; Gast et al. 1983; Poluektov et al. 2005), and the triplet signal slowly decreases in intensity during the light-induced ENDOR experiment with a rate that depends on the illumination conditions. Under our conditions, after ≈ 14 h of measurements the ^3^P700 signal was too weak to continue acquisition, and a new sample was required to continue. The acquisition time for one ENDOR spectrum shown in Fig. 4 at the canonical Z orientation was about 10–12 h.

The Q-band FSE-EPR spectrum of ^3^P700 in frozen PSI preparations at 30 K is similar to the one from ^3^P680 (see Figs. 4B vs 3B), showing the typical polarization pattern AEEAAE of a ^3^Chl generated via the RP (ST_0_ triplet) mechanism (Angerhofer 1991; Budil and Thurnauer 1991). As for ^3^P680, the pulse EPR spectra exhibit reduced signal intensities as compared to transient (direct detection) EPR spectra for non-canonical orientations (Lendzian et al. 2003). In addition to the echo-detected pulse spectra we have also recorded FID-detected pulse spectra, which are more similar to the transient EPR spectra, demonstrating that nuclear modulation effects are indeed one contributing factor to the differences between echo-detected pulse EPR spectra and transient EPR spectra (Fig. S8). In the Q-band EPR spectra, strong additional signals are observed in the *g* ≈ 2.00 region (≈ 1208 mT). Part of this signal is the light-induced radical pair P700^·+^A_1_^·−^ in the small fraction of RCs where the A_1_ was not reduced to A_1_^·−^ during the photoaccumulation procedure. The largest contributions to this region come from photoaccumulated A_1_^·−^ and from other stationary radicals like A_0_^·−^. Subtraction of the “dark” background spectrum works here only partially since the strong signals in the radical region (around *g* ≈ 2.00) were saturating the detection system. The high amplification leading to saturation of the strongest signals were chosen since we strived for sufficient dynamic range for the relatively weak triplet signals. Anyway, this has no effect on the triplet EPR spectrum (except for this narrow region) and no effect for the ENDOR measurements. The ZFS parameters (|D|= 0.0278 cm^−1^, |E|= 0.0038 cm^−1^) are in good agreement with several previous EPR and ODMR studies (Frank et al. 1979; Sieckmann et al. 1993; Vrieze et al. 1996; Carbonera et al. 1997; Santabarbara et al. 2002; Poluektov et al. 2002). They are slightly smaller than the ones from ^3^P680 (|D|= 0.0288 cm^−1^, |E|= 0.0043 cm^−1^), but lie in the typical range for monomeric chlorophylls. The ZFS parameters are collected in Table 1 together with the g tensor values and compared with those obtained for other ^3^Chl species. Note, that the g tensor values of ^3^P700 and ^3^P680 are almost identical. From the similarity of the ZFS values we conclude that at cryogenic temperature the triplet exciton is located on a monomeric chlorophyll also in ^3^P700, in agreement with the conclusions from the previous studies and fully confirmed by the observed hfc values in the ENDOR spectra described below.

In Fig. 4 the pulse Q-band ^1^H-ENDOR spectra of ^3^P700 at 30 K, recorded at all the canonical field positions, are also shown. The higher temperature in comparison to the measurement at 10 K on ^3^P680 was chosen to prevent the reduced [4Fe–4S]-clusters of PSI (F_X_, F_A_, F_B_), which gave EPR signals in test measurements at low temperatures (data not shown), from contributing to the ^1^H ENDOR signals that potentially overlap with those of ^3^P700. At 30 K the relaxation of these FeS-clusters is fast in comparison to the time required for performing the ENDOR experiment (> 10 µs). As for the previous ENDOR spectra, the frequency scale measures the deviations from the proton Larmor frequency, the ENDOR transitions are labeled as positive or negative with respect to their appearance in the spectrum relative to *ν*_H_, and the emissive ENDOR spectra have been inverted for straightforward comparison. The ^1^H hfcs and their assignments, together with the calculated isotropic hyperfine constants are reported in Table 2 and compared with the corresponding values for ^3^P680 and ^3^Chl *a* and the DFT calculations.

At the Z canonical orientation, three prominent ENDOR lines associated with negative hfcs can be observed. Since they are very similar to those in ^3^P680, we directly assign them to the methine protons **5**, **10,** and **20.** While their linewidth is similar to those in ^3^P680 for the Z orientation, their ENDOR lines at the X (and somewhat at Y) canonical orientations are broader and quite weak. We explain this by a rotation of the methine proton hyperfine tensors in the plane by ≈20° with respect to the ZFS tensor axes, which results in the broadening of the ENDOR signals at X orientation (see Fig. S5). Note, that we cannot distinguish from the simulation which of the tensors (ZFS or hfc) is rotated in the molecular frame. However, since there is no indication for a change in the local symmetry around the methine protons, a rotation of the ZFS tensor seems more likely. Interestingly, an LD-ODMR study of ^3^P700 and ^3^Chl *a* noted differences between these two with respect to the orientation of the ZFS axes in the molecular frame (Vrieze et al. 1996). They concluded that a sign inversion of E (exchange of the in-plane axes X and Y) does not take place, in full agreement with our conclusions based on the Q-band ENDOR data. They determined that the in-plane triplet axes are slightly rotated with respect to ^3^Chl *a*. This could explain the fact that our ^3^P700 α-proton signals are broader than those of ^3^P680 for the X (and Y) orientation. A substantially larger in-plane rotation can be excluded, since the ENDOR signals of the α-protons at X would become very broad, probably beyond detection. A 130 GHz EPR study on ^3^P700 and ^3^Chl *a* observed a reversal of g-value ordering for *g*_*X*_ and *g*_*Y*_ (*g*_*X*_ > *g*_*Y*_ for ^3^P700 and *g*_*Y*_ > *g*_*X*_ for ^3^Chl *a*) and interpreted this as a switching of the two in-plane ZFS axes X and Y (Poluektov et al. 2002). Our ENDOR data clearly contradict this interpretation: two strong negative hyperfine couplings were observed for the Y orientation, which are in the expected ratio to those observed at Z orientation (for both protons **10** and **20**, A_Y_/A_Z_ ≈ 1.5). If ZFS axes X and Y would be switched, the ratio should be closer to 0.5 than 1.5. Furthermore, the orientation dependence of the methyl **2** and **12** protons also contradicts this conclusion: both methyl groups show the largest hfc component at X orientation, which is in good approximation collinear to the C–CH_3_ bond of methyl groups **2** and **12,** exactly as expected (McConnell et al. 1960; Heller and McConnell 1960; Carrington and McLachlan 1969; Gordy 1980). The exact reason for the inversion of the g-tensor value ordering is thus not clear, maybe quite subtle electronic changes associated with the small rotation of the ZFS tensor are causing the small changes of *g*_*X*_ and *g*_*Y*_. This is certainly an interesting effect and would warrant a detailed investigation, probably using advanced computational approaches to disentangle the (various) causes leading to this effect and comparing it with computational work on ^3^P680 along the same lines.

The broadening makes the determination of the hfcs and the assignment of specific protons more challenging than for ^3^P680. A tentative assignment is reported in Table 2.

Important differences in terms of spectral characteristics of the primary donor triplet spectra have been found in the region where positive hfcs are detected. The ENDOR lines belonging to methyl protons at position **12**, which are narrow and intense not only in the case of ^3^P680 but also for ^3^Chl *a* in solution and the WSCP protein matrix, are detectable but broad and very weak for all canonical orientations in ^3^P700. On the other hand, the methyl group protons at position **2** contribute an intense ENDOR line at all canonical positions, as also found for ^3^Chl *a* in WSCP but in contrast to what we and Lendzian et al. (2003) observed for ^3^P680 in D1D2Cyt*b*_*559*_. Assignments of methyl protons at position **7** and the β protons at positions **17** and **18** are based on comparison with the ENDOR spectra of ^3^P680 and DFT calculations. While the assignment of the vinyl protons **3′** and **3″** is complete for ^3^P680, only the largest hfc component is visible in the ENDOR spectrum of ^3^P700 at the Y canonical orientation.

## Discussion

### Previous work on BChl triplets and bacterial reaction centers

The triplet states in the photosynthetic RCs and their model systems have been extensively investigated by time-resolved transient and pulse EPR techniques. To determine the electron-nuclear hyperfine couplings (hfcs) of protons, pulse ENDOR, combined with laser excitation, is the best-suited technique (Kulik and Lubitz 2009). The determination of the hfcs allows the most precise quantification of the extent of triplet delocalization and provides a means to assign the triplet to a specific chlorophyll. The technique has been extensively used to study the electron spin density distribution of the triplet state for porphyrin derivatives and (bacterio)chlorophylls and also for photosynthetic primary donors (Kay et al. 1995; Di Valentin et al. 1996; Lendzian et al. 1998, 2003; Marchanka et al. 2009; Tait et al. 2015; Richert et al. 2017; Agostini et al. 2017, 2019a, 2020; Barbon et al. 2020). The monomeric or dimeric nature of the special pair, and the influence of the protein surroundings on the excitation sharing, could be assessed by mapping the distribution of the unpaired electrons in the molecular system ^3^P865 and ^3^P960 in the bacterial RCs of *Rhodobacter sphaeroides* and *Blastochloris viridis*, respectively. Delocalization was demonstrated on the basis of a comparison of the ENDOR spectra with those recorded on the triplets of bacteriochlorophyll *a* and *b *in vitro (Marchanka et al. 2009) and confirmed later based on highly resolved ENDOR data at 34 GHz and comparison with DFT calculations on the bacteriochlorin macrocycle (Marchanka et al. 2014). Furthermore, triplet–triplet transfer to a carotenoid in the RC could be demonstrated in this work (Marchanka 2009; Marchanka et al. 2009). Such detailed information is still missing for ^3^P700 in PSI, and incomplete for ^3^P680 in PSII (Di Valentin et al. 1996; Lendzian et al. 2003). In the following we want to discuss the experiments done on both states, draw conclusions on the electronic structure, propose the localization of the triplet state and relate this to function.

### Assignment and localization of the triplet state from EPR data

The FSE-EPR spectra of ^3^P680 and ^3^P700 at cryogenic temperatures presented in this work both show the same spin polarization pattern AEEAAE and the analyses give very similar ZFS and g tensor parameters (see Table 1). Furthermore, these data agree well with those obtained for monomeric chlorophyll *a* triplet states in solution or embedded in a protein matrix (Poluektov et al. 2002; Lendzian et al. 2003; Di Valentin et al. 2009; Agostini et al. 2017). As discussed earlier this shows that ^3^P680 and ^3^P700 are created via RP recombination in the PSII and PSI reaction centers following the initial charge separation and form so-called ST_0_ triplets. The ZFS values suggest that the two triplet states are localized on monomeric chlorophyll molecules at low temperatures. This conclusion is corroborated by the measured ^1^H hfcs obtained in this work for all major α- and β-protons (Table 2).

### Triplet delocalization on more than one species from ENDOR data at low temperature

Our ENDOR spectra, with the expected number of lines and the lines not showing any sign of splitting, further demonstrate that the triplet exciton is localized on a specific single Chl at low temperatures, and not on different Chls in different RC fractions. Indeed, the hfcs are similar for ^3^P680, ^3^P700, and ^3^Chl monomer in vitro and in WSCP. If slow hopping (slow with respect to the time scale of the ENDOR experiment) between two chlorophylls would occur, we would observe a splitting of the ENDOR lines since the central chlorophylls in the RC of PSI and PSII are not electronically equivalent (see Fig. 1). If there would be delocalization over more than one Chl or fast hopping (fast with respect to the time scale of the ENDOR experiment) between two chlorophylls, the hfcs would be reduced to about half in case of an equally shared triplet exciton or, for unequal sharing, the total number of lines would be increased and show smaller hfcs for one Chl and larger for the other Chl, reflecting the relative spin populations on the respective chlorophylls. The sum of the hfcs from both Chls would approximately equal the ones of the monomeric ^3^Chls. These cases can thus with certainty be excluded both for ^3^P680 and ^3^P700.

### Comparison with DFT calculations

Measurements of the hfcs using ENDOR also offer a means to probe the heuristic values of modern quantum chemical calculations on these systems. We have included in Table 2 calculations that were performed on monomeric ^3^Chl *a* using DFT (Agostini et al. 2019a). The comparison of the assigned experimental hfcs in the various systems with those calculated on the model system shows very good agreement for the methyl proton hfcs (positions **2**, **7**, **12**) probing different regions of the macrocycle. The methine α-protons (positions **5**, **10**, **20**) are somewhat underestimated, which is a known problem for DFT calculations on tetrapyrrole systems like Chls or BChls (Sinnecker and Lubitz 2017). The vinyl group protons are reproduced quite well; the values for the β-protons at positions **17**/**18** are satisfactory calculated, they depend strongly on the correct dihedral angles. The information on the hfcs is important since the related spin density distribution of the triplet state yields a picture of the electronic distribution in the frontier orbitals of the system that otherwise cannot be obtained. This information on the electronic structure is crucial for a theoretical understanding of the primary processes in PSI and PSII involving P700 and P680 in charge separation and recombination as well as exciton transfer.

### Triplet delocalization determined from EPR at higher temperatures

The temperature-dependence of the delocalization extent of the primary donor triplet exciton in PSI and PSII was previously investigated by time-resolved EPR at X-band and in some cases also at higher fields (Sieckmann et al. 1993; Bosch et al. 1996; Kamlowski et al. 1996; Frankemöller et al. 1998; Pashenko et al. 2003; Niklas 2007). Different interpretations of the temperature-dependence of the triplet state spectrum of ^3^P680 have been given, among them delocalization at higher temperatures of the triplet exciton that is at cryogenic temperatures located on the Chl_D1_, populating in addition the chlorophyll P_D1_ and eventually also P_D2_, the pheophytin Pheo_D1_ and possibly also the second accessory Chl_D2_ (see Fig. 1B). For ^3^P700, the temperature-dependence of the time-resolved EPR was interpreted as a triplet exciton that is delocalized over the two halves of the Chl dimer P_A_ and P_B_ at room temperature while the triplet exciton is trapped on one half at low temperature (below 50 K) (Sieckmann 1993; Niklas 2007). The temperature dependence of both ^3^P680 and ^3^P700 was investigated in (Niklas 2007) and the earlier work of Stehlik and coworkers was confirmed (Sieckmann et al. 1993; Kamlowski et al. 1996). In the interpretation of these data a complication arises because information from the ZFS parameters cannot be unequivocally interpreted in terms of delocalization because of possible effects caused by the charge transfer character of the triplet state and/or molecular distortions of the macrocycle, as highlighted in the case of porphyrin model systems (Tait et al. 2015; Bolzonello et al. 2017). Here the determination of the hfcs using ENDOR is very helpful, but ENDOR on triplet states at elevated temperatures is very difficult due to fast spin relaxation, in particular for difficult cases as ^3^P700 (Sieckmann et al. 1993; Niklas 2007).

### Assignment of triplet states in PSI and PSII

For the assignment of the (quasi-monomeric) ^3^Chls observed in our experiments to a specific Chl we have adopted an approach that is based on the impact of the protein surrounding on the cofactor(s). The important role of the pigment-protein interactions has clearly emerged in most spectroscopic characterizations of photosynthetic cofactors, as demonstrated, for example, in the investigations on the effects of point mutations (Rautter et al. 1995; Schulz et al. 1998; Krabben et al. 2000; Webber and Lubitz 2001; Witt et al. 2002; Müh et al. 2002; Lubitz 2006). In the present work, this aspect can be analyzed in detail by comparing ENDOR results of the same species (^3^Chl *a*) in different protein surroundings as hfcs are sensitive probes of the local environment of the nuclei under investigation. In the specific case of the primary donor, this is remarkably important, considering that the debate on the site of triplet localization is still not resolved, although EPR spectroscopy and optical methods have been extensively used to address this issue (Rutherford and Sétif 1990; van Mieghem et al. 1991; Zech et al. 1999; Krabben et al. 2000; Diner et al. 2001; Breton 2001; Kammel et al. 2003; Kawamori et al. 2005; Zabelin et al. 2016; Takegawa et al. 2019). Even though the direct comparison of the triplet ENDOR spectra of chlorophylls reveals similar hyperfine patterns (Table 2), some signals are clearly affected by the specific environment of the Chl *a* species.

From the comparison of the well resolved ^1^H-ENDOR of ^3^Chl *a* in various Chl-binding proteins (PSI, PSII, and WSCP, see Table 2), it clearly appears that while the signals for the three methine α protons (**5**, **10**, **20**) are usually intense and observed in triplet ENDOR (Di Valentin et al. 1996; Lendzian et al. 2003; Agostini et al. 2017, 2019a, 2020), the signals of the β protons of the three methyl groups (**2**, **7**, **12**) have an intensity that seems to significantly vary in the investigated protein complexes, leading to the difficulty to detect methyl group **2** in ^3^P680 (D1D2Cyt*b*_*559*_) (Fig. 3) and the massive weakening and broadening of methyl group **12** in ^3^P700 (Fig. 4). These findings point towards a marked sensitivity of the methyl proton peak intensities to the different protein binding sites of the ^3^Chls in the two photosystems. A lowering of the intensity and a concomitant broadening of the methyl peaks is commonly explained in terms of hindered rotation of the methyl group, as previously observed for example in bacteriochlorophyll *a* (Feher et al. 1975)*.* In order to evaluate the steric hindrance exerted from the binding sites to the methyl groups of interest, we analyzed their chemical environment in terms of the number of atoms in a 4 Å radius from each methyl carbon atom (see Fig. S10). For this contact analysis, we focused our attention on the chlorophylls constituting the P680 and P700 species as well as those adjacent to them (see Fig. 1A and B). WSCPs are symmetric homotetrameric complexes (Horigome et al. 2007; Bednarczyk et al. 2016; Agostini et al. 2019b), in which the four present Chls are bound to identical binding sites in each of the two WSCPs previously investigated (Agostini et al. 2017, 2020). Thus, the analysis of just one binding site is sufficient.

From a comparison of the number of contacts between the different Chl binding sites, it appears that those of the two WSCPs are characterized by a low number of contacts in the vicinity of all three methyl groups, in good agreement with the fact that the ENDOR lines of the three freely rotating methyl groups can easily be observed in these two systems (Agostini et al. 2017, 2019a, 2020).

When the number of contacts of the methyl group **12** is compared between all the analyzed Chl binding sites, it clearly appears that the P_A_ site of P700 is characterized by a marked steric encumbrance generated by four amino acids surrounding this methyl group (two phenylalanines, one leucine and one alanine). The strong asymmetry of this particular zone in P_A_ and P_B_ (binding of a Chl *aʹ* in P_A_) is determined by a different protein structure for the two Chl-binding sites in the surrounding of ring E (Jordan et al. 2001) adjacent to the pyrrole to which methyl **12** is bound. This could explain the marked difference in the number of contacts of methyl **12** between the binding sites of P_A_ and P_B_. A localization of ^3^P700 on P_A_ could therefore be concluded from this analysis. This is in agreement with previous FTIR data (Breton 2001, 2006) but disagrees with earlier data from EPR and ODMR obtained from mutant studies (Webber and Lubitz 2001; Lubitz 2006). This discrepancy shows that the triplet localization/delocalization is not yet finally solved and requires further spectroscopic experiments on mutants and other PSI preparations, and also theoretical work – including advanced quantum chemical studies that are underway in our laboratories.

The difficulty to detect the methyl peak at position **2** in ^3^P680 (in D1D2Cyt*b*_*559*_) was proposed in (Lendzian et al. 2003) to be caused by the neighboring vinyl group that would lead to its clash with the neighboring methyl group. However, WSCPs from different organisms were found to have methyl **2** peaks of comparable intensity (Agostini et al. 2017, 2020), despite the corresponding X-ray structures revealed that the bound Chls display opposite vinyl configurations (Horigome et al. 2007; Bednarczyk et al. 2016). The appearance of the methyl group **2** resonances seems to depend on the PSII preparation. Whereas no signal could be detected for the D1D2Cyt*b*_*559*_ complex, the methyl **2** resonances clearly showed up in the PSII core complex preparation (with doubly reduced Q_A_; S. Prakash, J. Niklas, and W. Lubitz, manuscript in preparation). It is known that the biochemical isolation of the D1D2Cyt*b*_*559*_ complex perturbs the surrounding of ^3^P680 (Carbonera et al. 1994) and P680^**·+**^ (Okubo et al. 2007; Krausz et al. 2008; Acharya et al. 2012), therefore the crystal structure of PSII (Umena et al. 2011) is expected to be close to the one of the PSII core structure, whereas in the case of the D1D2Cyt*b*_*559*_ complex deviations can be expected. The localization of ^3^P680 on Chl_D1_ (Diner et al. 2001; Kawamori et al. 2005; Zabelin et al. 2016; Takegawa et al. 2019) is in good agreement with the low number of contacts of the three methyl groups displayed by both Chl_D1_ and Chl_D2_. From the disappearance of the methyl **2** peak in the D1D2Cyt*b*_*559*_ complex, it can be assumed that in this complex a structural perturbation close to Chl ring A induces a steric crowding in the proximity of methyl **2**. Another intriguing possibility is that the triplet state sits on different Chls in the two PSII complexes.

We have also investigated if a carotenoid is close to any of the chlorophylls in the RC of PSII that could carry the triplet state (Chl_D1_, Chl_D2_, P_D1_, P_D2_). Although there are several Car species present (Umena et al. 2011) none of these seems to be close enough to allow efficient triplet–triplet energy transfer from ^3^Chl to Car to eliminate the dangerous triplet exciton. Close contact of carotenoids to the primary donor of PSII is also not likely since the very high oxidation potential of > + 1.2 V of P680/P680^**·+**^ would lead to oxidation and degradation of the carotenoid (Telfer 2002). It is known that in PSII the formation of ^3^Chl causes formation of singlet oxygen ^1^O_2_ (Krieger-Liszkay et al. 2008) via reaction with the triplet oxygen ^3^O_2_ released by the water oxidizing complex (Mn_4_O_5_Ca) in PSII (Lubitz et al. 2019). The singlet oxygen leads to degradation of the D1 protein of PSII, which has only a lifetime of ≈ 30 min. A repair cycle is in place in all photosynthetic organisms to reconstitute the D1 protein and thus keep the water splitting cycle and PSII intact (Nixon et al. 2010). It is quite clear that in the central D1D2 protein of PSII the formed ^3^Chls cannot be effectively quenched by carotenoids as in other photosynthetic proteins, e.g., the antenna systems. The very special situation of PSII in oxygenic photosynthesis left *Nature* no other choice than to develop a highly efficient repair cycle for this central protein.

## Conclusions

In this comparative work, we have performed for the first time a comprehensive ^1^H-ENDOR characterization at Q-band of the triplet states ^3^P680 in PSII and ^3^P700 in PSI reaction centers. ^1^H-ENDOR measurements at all ZFS canonical orientations have allowed us to obtain and assign a complete set of the large hfcs (> 1 MHz) of the α and β protons bound to the Chl macrocycle. The experimental assignment of the full set of measured couplings to specific molecular positions is in agreement with DFT calculations, based on a computational approach, which has been optimized in a previous investigation on ^3^Chl *a* in WSCP and provides reliable hfc tensors (Agostini et al. 2019a). The large positive hfcs are attributed to two different couples of rotating methyl group (β) protons, which alternate in intensity in the two photosystems, while the more anisotropic negative couplings are attributed to methine (α) protons attached directly to the macrocycle.

This complete picture of the proton hyperfine interactions has been interpreted in terms of localization on a single, specific Chl unit, at low temperatures, both for ^3^P700 and for ^3^P680. Rationalization of the effects on the ENDOR spectra produced by differences in the binding site has provided evidence for the localization of ^3^P700 on P_A_ (see Fig. 1). At higher temperatures a delocalization of the triplet exciton has been proposed based on transient and pulse EPR data performed on the triplet states in both PSI and PSII (Sieckmann et al. 1993; Kamlowski et al. 1996; Frankemöller et al. 1998; Niklas 2007), which could be corroborated in our EPR experiments (data not shown). However, ENDOR experiments at elevated temperatures are very difficult to perform due to fast relaxation.

The precise hyperfine couplings and spin density distributions for ^3^P680 and ^3^P700 obtained in this work provide a solid basis for a detailed future comparison with state-of-the-art quantum chemical calculations on high-resolution structures of PSII and PSI. This approach promises also to be successful for a final assignment of the triplet state to specific Chls in the two photosystems. The precise spin density distribution on the primary donor is also important for a sound general theoretical understanding of the electron transfer processes as well as for triplet–triplet energy transfer, to guarantee efficient photoprotection, even if this is not important in the particular case of the primary donor of PSII and PSI.

In perspective, detailed knowledge of the factors governing the extent of triplet state localization and delocalization is also important for the optimization of the photophysical processes in devices for applications in solar energy conversion, molecular electronics and spintronics. A “learning from nature” approach, in the specific case of the excited triplet state, can take advantage of the information on the electronic structure directly derived for the paramagnetic endogenous probe by hyperfine spectroscopy.

## Supplementary Information

Below is the link to the electronic supplementary material.

Supplementary file1 (PDF 1211 kb)
