# Supplementary material for: Primary donor triplet states of Photosystem I and II studied by Q-band pulse ENDOR spectroscopy
Source: Photosynth Res. 2022 Mar 15;152(2):213–34. doi: 10.1007/s11120-022-00905-y (PMC9424170; doi:10.1007/s11120-022-00905-y)
Supplement: Supplementary file 1 — Supplementary file1 (PDF 1211 kb) [file 11120_2022_905_MOESM1_ESM.pdf]

Supporting Information

Primary Donor Triplet States of Photosystem I and II Studied by Q-Band Pulse ENDOR Spectroscopy

Jens Niklas[1,2,5,*], Alessandro Agostini[3,4,5], Donatella Carbonera[3], Marilena Di Valentin[3,*], Wolfgang Lubitz[1,*]

[1] Max Planck Institute for Chemical Energy Conversion, Stiftstrasse 34-36, 45470 Mülheim an der Ruhr, Germany

[2] Current address: Chemical Sciences and Engineering Division, Argonne National Laboratory, 9700 S. Cass Ave., Lemont, IL, 60439, USA

[3] Department of Chemical Sciences, University of Padova, via Marzolo 1, 35131 Padova, Italy

[4] Biology Centre, Institute of Plant Molecular Biology, Czech Academy of Sciences, Branišovská 31, 370 05, Ceske Budejovice, Czech Republic

[5] These authors equally contributed

***** Correspondence should be addressed to J.N. (email: jniklas@anl.gov), M.D.V. (email: marilena.divalentin@unipd.it) or WL (email: wolfgang.lubitz@cec.mpg.de).

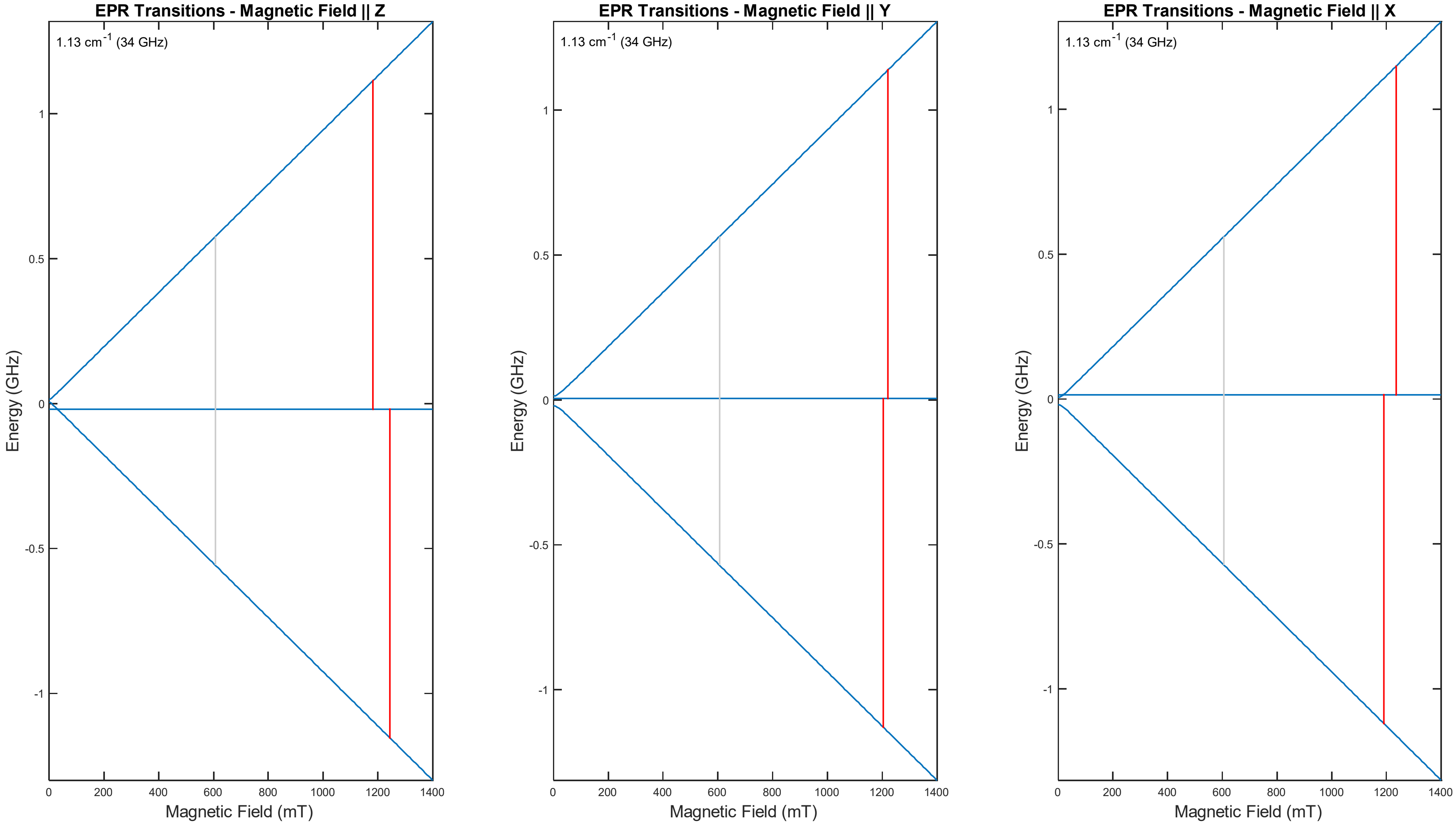

Fig. S1. Triplet spin energy levels as function of an external magnetic field along the canonical orientations X, Y, and Z. The Zero-Field Splitting (ZFS) parameters D and E are typical in sign and magnitude for monomeric chlorophyll triplet states (D= +863 MHz (≈0.0288 cm^{-1}), E=-129 MHz (≈0.0043 cm^{-1})). EPR transitions at Q-band (here: 34 GHz) are indicated by vertical lines; allowed ΔM_S=1 transitions are indicated in red, forbidden ΔM_S=2 (half field) transitions are indicated in gray. Plots generated using EasySpin (Stoll & Schweiger 2006) in Matlab (The Mathworks, Inc., Natick, MA).

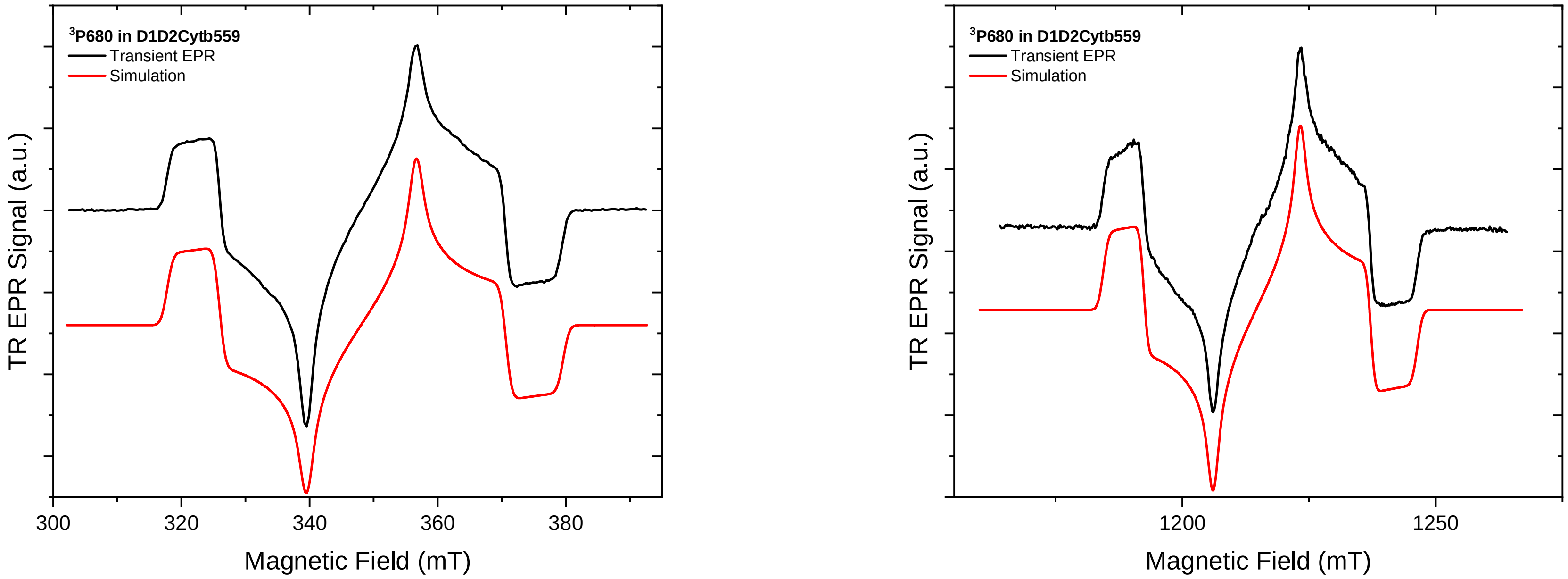

Fig. S2. Transient (direct detection) EPR Spectra of the ST_0 spin-polarized triplet ^{3}P680 in D1D2Cytb_{559} complexes from PSII (spinach) and their simulation. **Left panel.** Transient EPR Spectrum at X-band. **Right panel.** Transient EPR Spectrum at Q-band. Simulation parameters for both X- and Q-band: D = +288 ± 2 x10^{-4} cm^{-1}, E = -43 ± 2 x10^{-4} cm^{-1}, g_X=2.0031(±)0.0002, g_Y=2.0032(±)0.0002, g_Z=2.0022(±)0.0002. ZFS- and g-tensor principal axes are collinear. Absolute error in g-values is larger. Simulations generated using EasySpin (Stoll & Schweiger 2006) in Matlab (The Mathworks, Inc., Natick, MA).

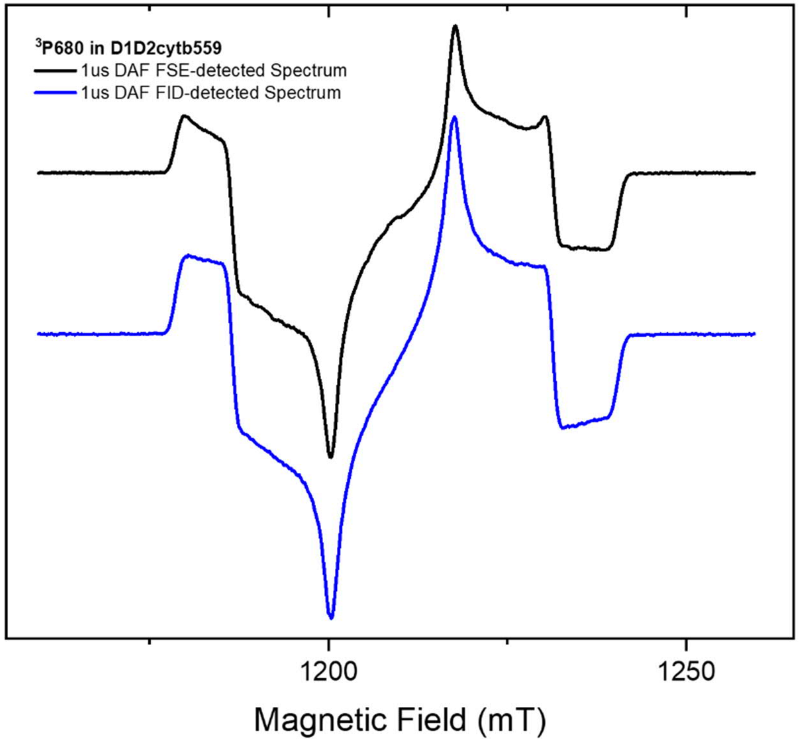

Fig. S3. Pulsed Q-band EPR Spectra of the ST_0 spin-polarized triplet ^{3}P680 in D1D2Cytb_{559} complexes from PSII (spinach) with DAF=1μs. FSE-detected EPR spectrum (black) and FID-detected EPR spectrum (blue), both corrected for the dark background signals. The FID-detected EPR spectrum exhibits a worse S/N ratio, but nuclear modulations effects are suppressed. The FID-detected pulse EPR spectrum is more similar to the transient EPR spectrum (Fig. S2), demonstrating that nuclear modulation effects are indeed one contributing factor to the differences between echo-detected pulse EPR spectra and transient EPR spectra (Figs. S2 and S3).

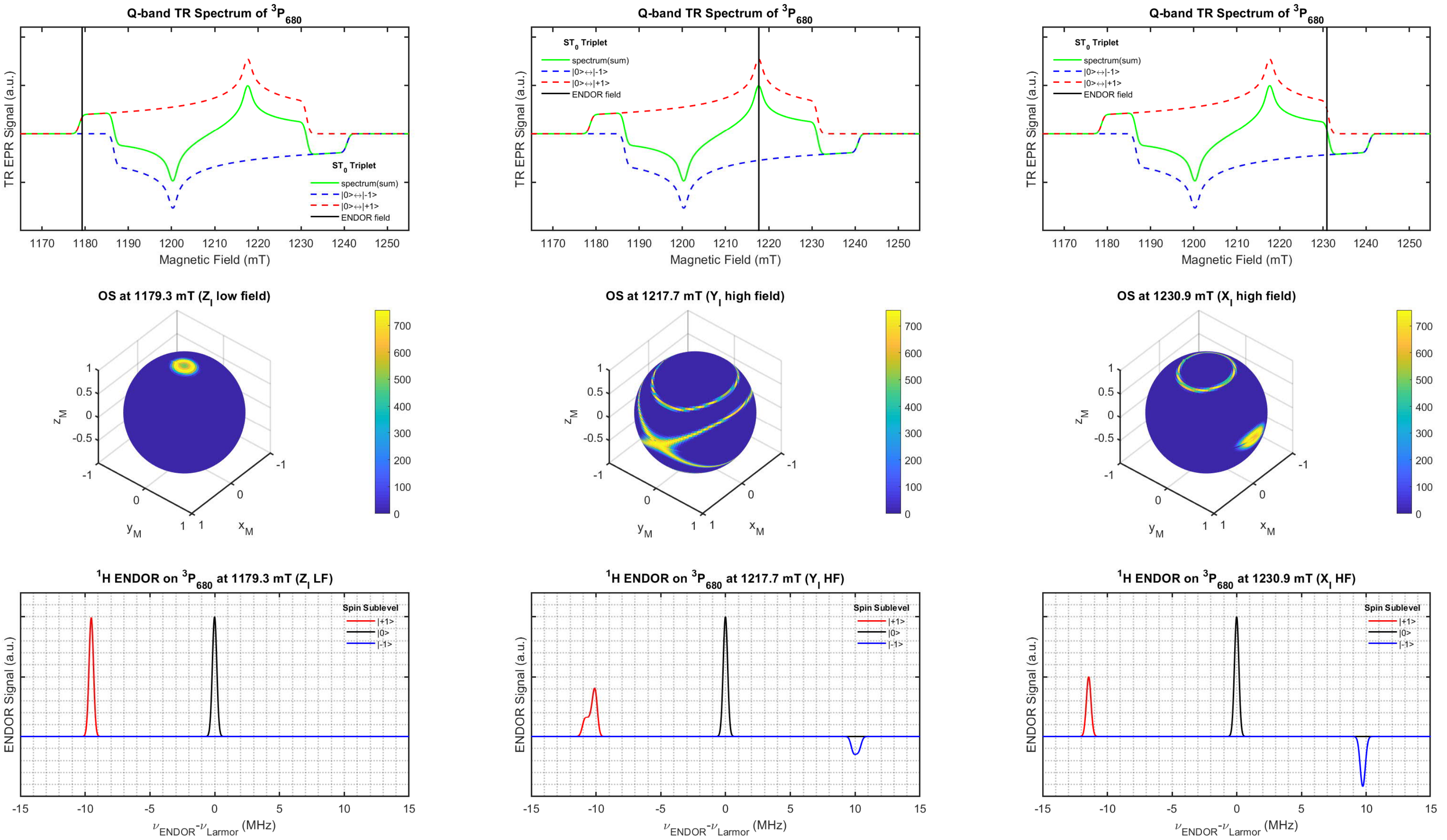

Fig. S4. Scheme of EPR and ENDOR spectroscopy for a ST_0 spin-polarized triplet chlorophyll (g ≈ 2.003, D = +869 MHz (≈0.0290 cm^{-1}), E = -128 MHz (≈0.0043 cm^{-1})). **Top panel.** Typical TR EPR spectrum of ^3P (like $^3P680$ or $^3P700$) at Q-band with magnetic fields indicated where ENDOR spectra were recorded. **Middle panel.** Orientation selection for ENDOR spectra recorded at the three magnetic fields corresponding to $Z_|$ low field, $Y_|$ high field and $X_|$ high field. **Bottom panel.** Simulated ^{1}H ENDOR signals of the spin-polarized triplet state for strongly coupled freely rotating methyl group protons (A_X = +11.5 MHz, A_Y = +10.0 MHz, A_Z = +9.6 MHz). Principal axes of the hyperfine tensor are

assumed to be collinear with ZFS-tensor and g-tensor. The intense and narrow ENDOR line at the Larmor frequency (black) stems from nuclear spin transitions in the $|T_0>$ sublevel, and ENDOR signals stemming from nuclear spin transitions in the $|T_{+1}>$ sublevel appear at frequencies lower than the Larmor frequency (red). ENDOR signals from nuclear spin transitions in the $|T_{-1}>$ sublevel appear at frequencies higher than the Larmor frequency (blue). The latter are only visible at $Y_|$ high field and $X_|$ high field since they are connected to $|T_0>\leftrightarrow|T_{-1}>$ electron spin transitions and thus show emissive ENDOR signals. Simulations generated using EasySpin (Stoll & Schweiger 2006) in Matlab (The Mathworks, Inc., Natick, MA).

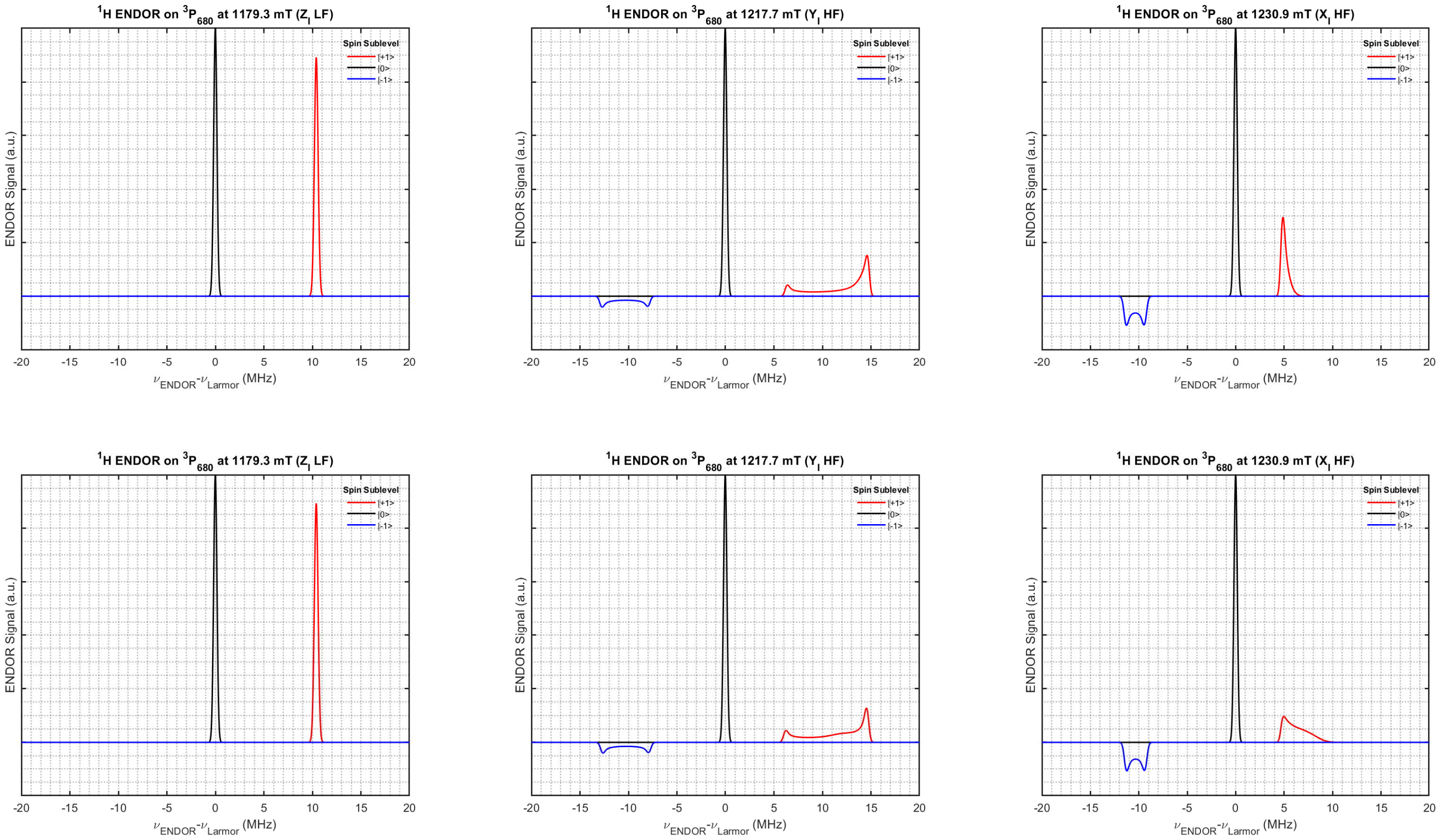

Fig. 5. Simulated ^{1}H ENDOR spectra for a ST_0 spin-polarized triplet chlorophyll (g ≈ 2.003, D = +869 MHz (≈0.0290 cm^{-1}), E = -128 MHz (≈0.0043 cm^{-1})). **Top panel.** Simulated ^{1}H ENDOR signals in the spin-polarized triplet state for strongly coupled methine α-proton (A_X = -4.7 MHz, A_Y = -14.8 MHz, A_Z = -10.4 MHz). Principal axes of the hyperfine tensor are assumed to be collinear with ZFS-tensor and g-tensor. **Bottom panel.** Simulated ^{1}H ENDOR signals in the spin-polarized triplet state for the same strongly coupled α-proton (A_X = -4.7 MHz, A_Y = -14.8 MHz, A_Z = -10.4 MHz). The hyperfine tensor has been rotated by 20° with respect to the ZFS-tensor and g-tensor in the (XY) plane. Since the α-proton hyperfine tensor is very anisotropic, even a 20° rotation has a pronounced effect on the ENDOR spectrum recorded at X orientation. Simulations generated using EasySpin (Stoll & Schweiger 2006) in Matlab (The Mathworks, Inc., Natick, MA).

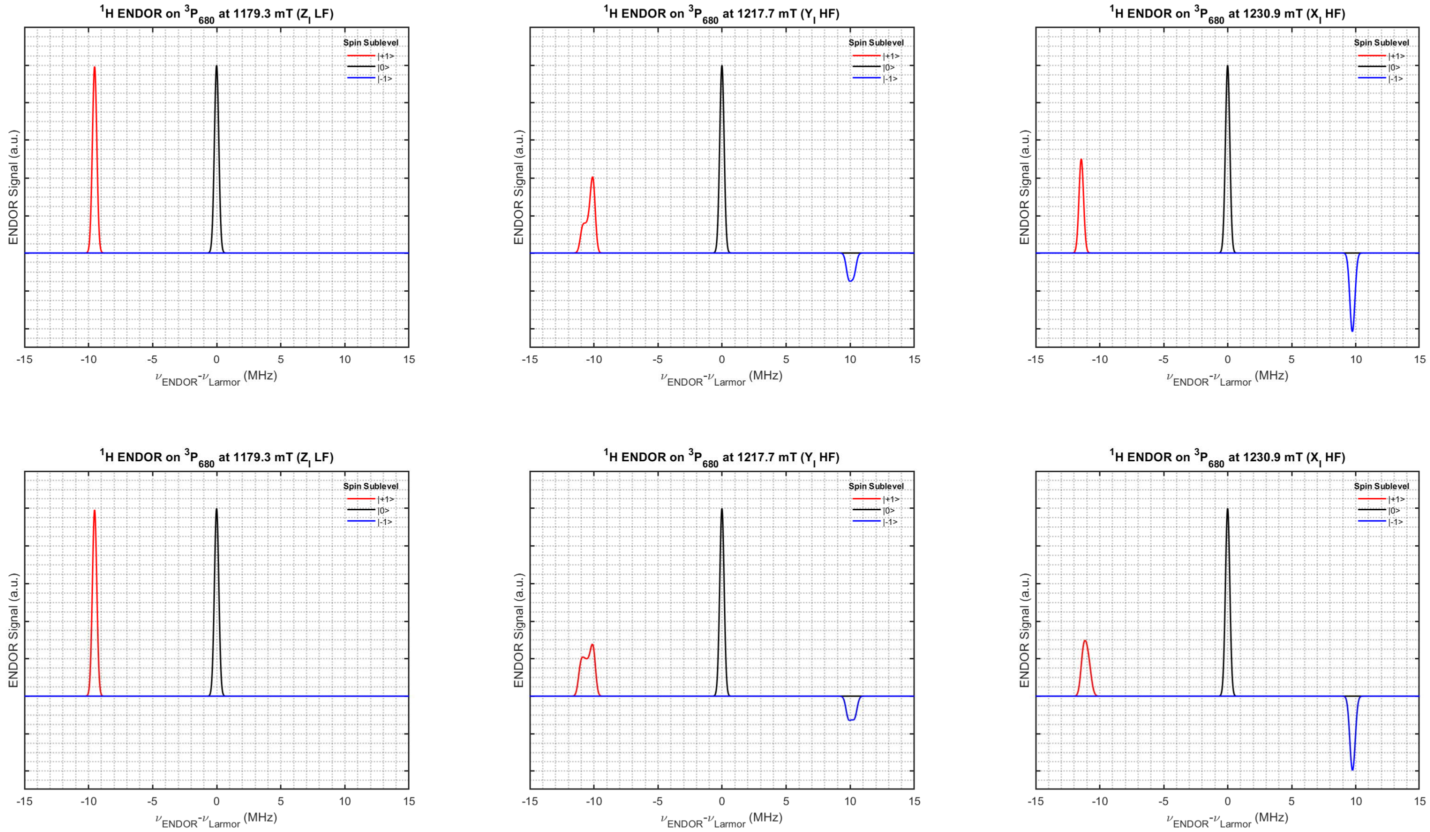

Fig. S6. Simulated ^{1}H ENDOR spectra for a ST$_0$ spin-polarized triplet chlorophyll (g ≈ 2.003, D= +869 MHz (≈0.0290 cm^{-1}), E=-128 MHz (≈0.0043 cm^{-1})). **Top panel.** Simulated ^{1}H ENDOR signals in the spin-polarized triplet state for strongly coupled freely rotating methyl group protons (A_X = +11.5 MHz, A_Y = +10.0 MHz, A_Z = +9.6 MHz). Principal axes of the hyperfine tensor are assumed to be collinear with ZFS-tensor and g-tensor. Same as bottom panel of Figure S4. **Bottom panel.** Simulated ^{1}H ENDOR signals in the spin-polarized triplet state for the same strongly coupled freely rotating methyl group protons (A_X = +11.5 MHz, A_Y = +10.0 MHz, A_Z = +9.6 MHz). The hyperfine tensor has been rotated by 30° with respect to the ZFS-tensor and g-tensor in the (XY) plane. Since the methyl group ^{1}H hyperfine tensor is quite isotropic, only subtle changes in frequencies and intensities are observed. Simulations generated using EasySpin (Stoll & Schweiger 2006) in Matlab (The Mathworks, Inc., Natick, MA).

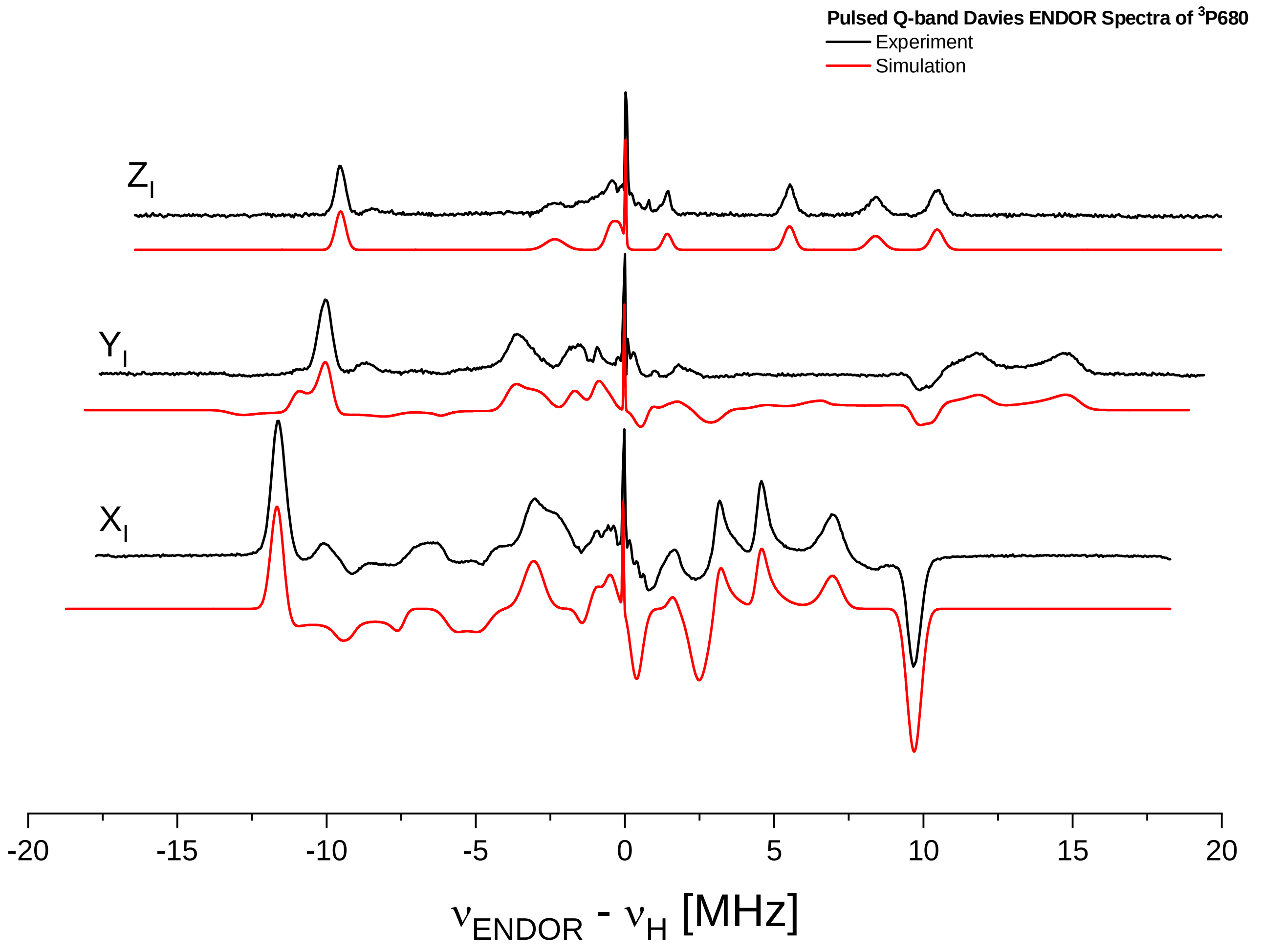

Fig. S7. Pulsed Q-band ^{1}H Davies ENDOR spectra of the ST_0 spin-polarized triplet ^{3}P680 in D1D2Cytb_{559} complexes from PSII (spinach) with DAF = 1μs and their simulation. Experimental spectra recorded at canonical positions $Z_|$, $Y_|$, and $X_|$ (black), and simulations (red). For simulation parameters, see Table 2. Simulations generated using EasySpin (Stoll & Schweiger 2006) in Matlab (The Mathworks, Inc., Natick, MA).

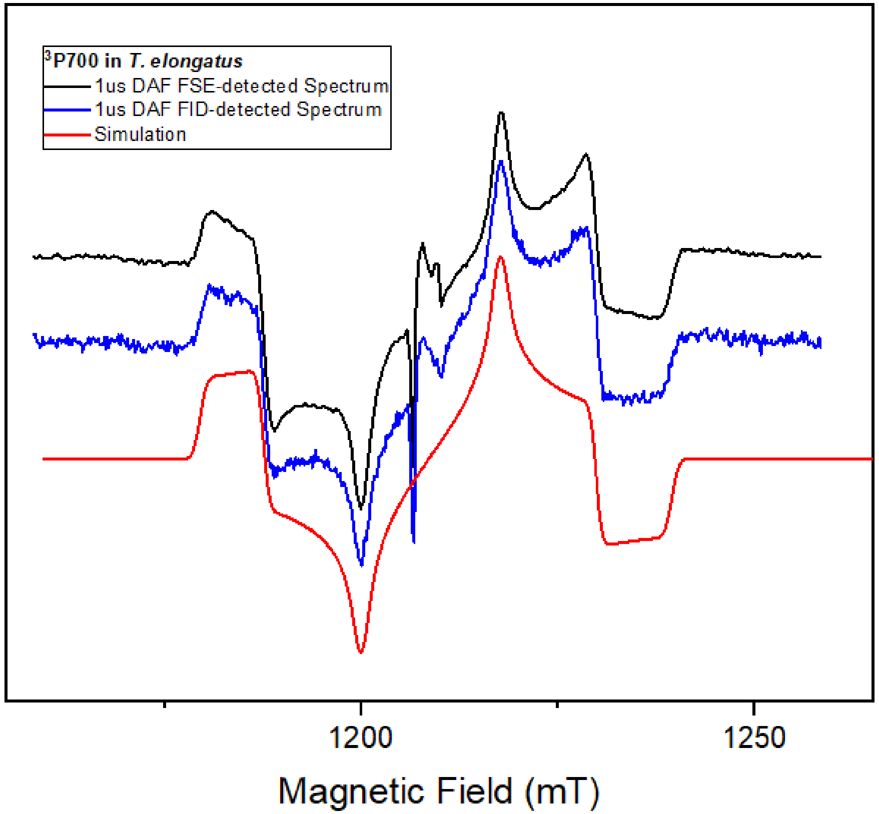

Fig. S8. Pulsed Q-band EPR spectra of the ST_0 spin-polarized triplet ^{3}P700 in PSI from *T. elongatus* with DAF = 1 µs and simulation (red). FSE-detected EPR spectrum (black) and FID-detected EPR spectrum (blue), both corrected for the dark background signals. The FID-detected EPR spectrum exhibits a worse S/N ratio, but nuclear modulations effects are suppressed. Simulation parameters: D = +278 ± 2 x10^{-4} cm^{-1}, E = -38 ± 2 x10^{-4} cm^{-1}, g_X = 2.0033(±)0.0002, g_Y = 2.0030(±)0.0002, g_Z = 2.0021(±)0.0002. ZFS and g-tensor principal axes are collinear. Absolute error in g-values is larger. Simulations generated using EasySpin (Stoll & Schweiger 2006) in Matlab (The Mathworks, Inc., Natick, MA).

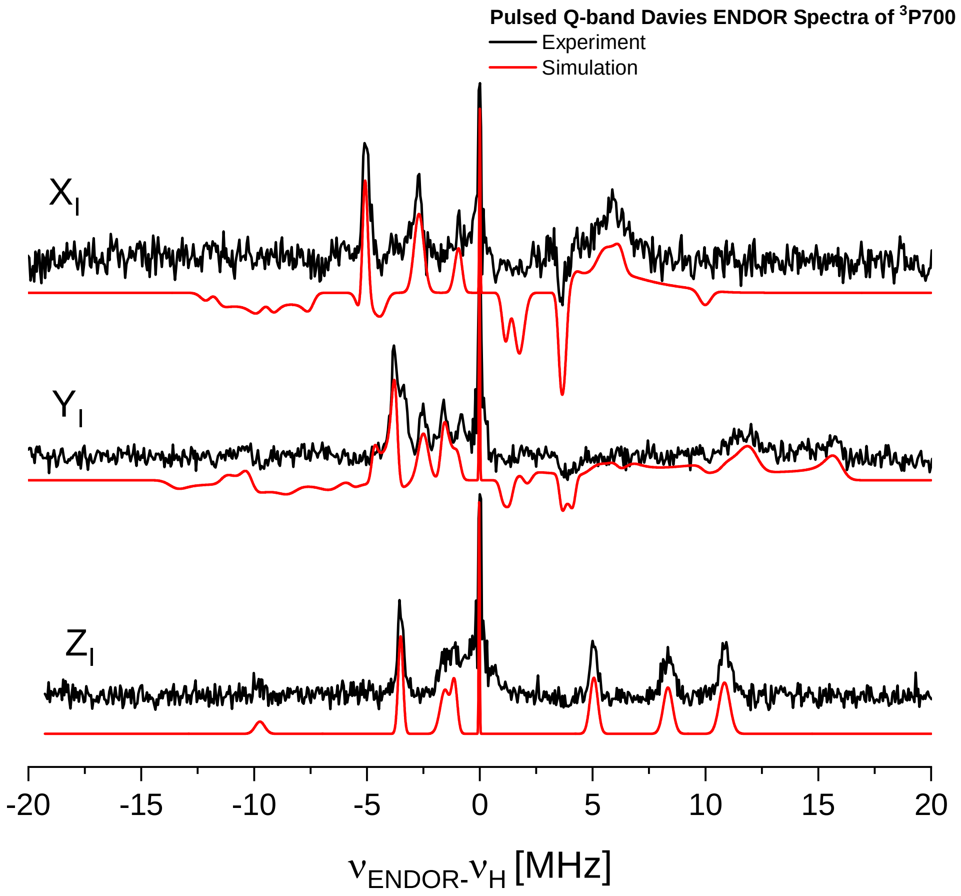

Fig. S9. Pulsed Q-band ^{1}H Davies ENDOR spectra of the ST_0 spin-polarized triplet ^{3}P700 in PSI from *T. elongatus* and their simulations. Experimental spectra recorded at canonical positions $Z_{|}$, $Y_{|}$, and $X_{|}$ (black), and simulations (red). For simulation parameters, see Table 2. Simulations generated using EasySpin (Stoll & Schweiger 2006) in Matlab (The Mathworks, Inc., Natick, MA).

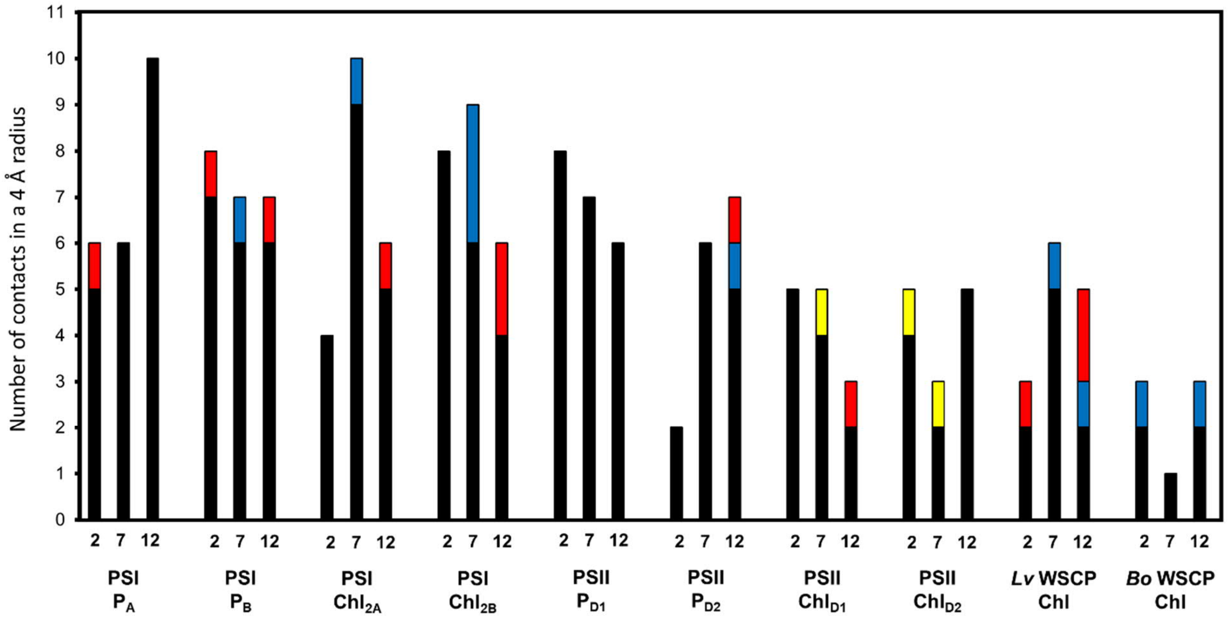

Fig. S10. Analysis of contacts in a 4 Å radius shell around the carbon atoms of methyl groups **2**, **7**, and **12** of the Chls of interest found in *T. elongatus* PSI (Jordan et al. 2001), *Chlamydomonas reinhardtii* PSII (Sheng et al. 2019), *Lepidium virginicum* (*Lv*) WSCP (Horigome et al. 2007), and *Brassica oleracea* (*Bo*) WSCP (Bednarczyk et al. 2016). Atoms belonging to the chlorin of the Chl itself have been excluded from the calculation. Carbon atoms are shown in black, oxygen atoms in red, nitrogen atoms in blue, and sulfur atoms in yellow. Hydrogen atoms, which are not present in the crystallographic structures, have not been counted.

References

Bednarczyk D, Dym O, Prabahar V, et al (2016) Chlorophyll Fine Tuning of Chlorophyll Spectra by Protein-Induced Ring Deformation. Angew Chemie Int Ed 55:1–6. https://doi.org/10.1002/anie.201512001

Horigome D, Satoh H, Itoh N, et al (2007) Structural mechanism and photoprotective function of water-soluble chlorophyll-binding protein. J Biol Chem 282:6525–6531. https://doi.org/10.1074/jbc.M609458200

Jordan P, Fromme P, Witt HT, et al (2001) Three-dimensional structure of cyanobacterial photosystem I at 2.5 Å resolution. Nature 411:909–917. https://doi.org/10.1038/35082000

Sheng X, Watanabe A, Li A, et al (2019) Structural insight into light harvesting for photosystem II in green algae. Nat Plants 5:1320–1330. https://doi.org/10.1038/s41477-019-0543-4

Stoll S, Schweiger A (2006) EasySpin, a comprehensive software package for spectral simulation and analysis in EPR. J. Magn. Reson.,178, 42-55. https://doi.org/10.1016/j.jmr.2005.08.013
